# Oncogenic PIK3CA recruits myeloid‐derived suppressor cells to shape the immunosuppressive tumour microenvironment in luminal breast cancer through the 5‐lipoxygenase‐dependent arachidonic acid pathway

**DOI:** 10.1002/ctm2.1483

**Published:** 2023-11-15

**Authors:** Xingchen Li, Guidong Chen, Fanchen Wang, Xiaojing Guo, Rui Zhang, Pengpeng Liu, Li Dong, Wenwen Yu, Huan Wang, Hailong Wang, Jinpu Yu

**Affiliations:** ^1^ Cancer Molecular Diagnostics Core, Tianjin Medical University Cancer Institute and Hospital National Clinical Research Center of Cancer, Tianjin's Clinical Research Center for Cancer Tianjin China; ^2^ Key Laboratory of Cancer Immunology and Biotherapy Tianjin China; ^3^ Key Laboratory of Breast Cancer Prevention and Therapy Tianjin Medical University, Ministry of Education Tianjin China; ^4^ Department of Thyroid and Neck, Affiliated Cancer Hospital of Zhengzhou University Henan Cancer Hospital Zhengzhou China; ^5^ Department of Breast Pathology and Lab, Tianjin Medical University Cancer Institute and Hospital, National Clinical Research Center for Cancer Tianjin Medical University Cancer Institute and Hospital Tianjin China; ^6^ Department of Immunology, Tianjin Medical University Cancer Institute and Hospital National Clinical Research Center for Cancer, Tianjin's Clinical Research Center for Cancer Tianjin China; ^7^ College of Life Sciences Nankai University Tianjin China; ^8^ Laboratory of Cancer Cell Biology, Tianjin Medical University Cancer Institute and Hospital National Clinical Research Center for Caner, Tianjin's Clinical Research Center for Cancer Tianjin China; ^9^ Key Laboratory of Cancer Prevention and Therapy Tianjin China

**Keywords:** arachidonic acid, luminal breast cancer, myeloid‐derived suppressor cells, PIK3CA, tumour immune microenvironment

## Abstract

**Background**: Oncogenic PIK3CA mutations (PIK3CA^mut^) frequently occur in a higher proportion in luminal breast cancer (LBC), especially in refractory advanced cases, and are associated with changes in tumour cellular metabolism. Nevertheless, its effect on the progression of the immune microenvironment (TIME) within tumours and vital molecular events remains veiled.

**Methods**: Multiplex immunohistochemistry (mIHC) and single‐cell mass cytometry (CyTOF) was used to describe the landscape of TIME in PIK3CA^mut^ LBC. The PIK3CA mutant cell lines were established using CRISPER/Cas9 system. The gene expression levels, protein secretion and activity of signaling pathways were measured by real‐time RT‐PCR, ELISA, immunofluorescence staining or western blotting. GSEA analysis, transwell chemotaxis assay, live cell imaging, flow cytometry metabolite analysis targeting arachidonic acid, Dual‐luciferase reporter assay, and Chromatin immunoprecipitation assay were used to investigate the underlying function and mechanism of the PI3K/5‐LOX/LTB4 axis.

**Results**: PIK3CA^mut^ LBC cells can induce an immunosuppressive TIME by recruiting myeloid‐derived suppressor cells (MDSCs) and excluding cytotoxic T cells via the arachidonic acid (AA) metabolism pathway. Mechanistically, PIK3CA^mut^ activates the transcription of 5‐lipoxygenase (5‐LOX) in a STAT3‐dependent manner, which in turn directly results in high LTB4 production, binding to BLT2 on MDSCs and promoting their infiltration. Since a suppressive TIME is a critical barrier for the success of cancer immunotherapy, the strategies that can convert “cold” tumours into “hot” tumours were compared. Targeted therapy against the PI3K/5‐LOX/LTB4 axis synergizing with immune checkpoint blockade (ICB) therapy achieved dramatic shrinkage in vivo.

**Conclusions**: The results emphasize that PIK3CA^mut^ can induce immune evasion by recruiting MDSCs through the 5‐LOX‐dependent AA pathway, and combination targeted therapy with ICB may provide a promising treatment option for refractory advanced LBC patients.

## Graphical abstract

Oncogenic PIK3CA^mut^ frequently occurs in a higher proportion in luminal breast cancer (LBC).

PIK3CA^mut^ initiated the arachidonic acid metabolism reprogramming by activating the 5‐LOX/LTB4 axis via STAT3, which further recruits myeloid‐derived suppressor cells infiltration and inhibits antitumour efficacy of T cells.

PI3K/5‐LOX/LTB4 axis sheds light on the promising future of synergistic treatment regimen of targeted therapy and immunotherapy for LBC patients.

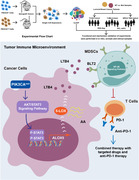

## 1 INTRODUCTION

Luminal tumours account for approximately two‐thirds of all breast cancers,^1^ and they have recently benefitted from a better prognosis than other subtypes of breast cancer on condition of the development of comprehensive therapeutic strategies.^2^ However, patients with advanced breast cancer still suffer from poor clinical outcomes due to drug resistance and tumour recurrence.^3^ In recent years, a number of genetic alterations in driver genes have been detected in refractory advanced luminal breast cancer (LBC), such as actionable mutations in PIK3CA, provoking the rapid development of targeted therapies in the clinic, including PI3K inhibitors.^4^, ^5^ However, significant disparity between clinical and preclinical data, and heterogeneity in objective response rates implied that a more complicated interaction network in the tumour immune microenvironment (TIME) might be involved in regulating the clinical efficacy of PI3K inhibitor‐based target therapies.^6^, ^7^, ^8^

Oncogenic mutations of PIK3CA (PIK3CA^mut^) are signature mutations in multiple cancers, which are present in approximately 30% of human breast cancer patients,^9^ and at a higher proportion in LBC patients,^8^ especially in refractory advanced disease.^10^, ^11^, ^12^ PIK3CA^mut^ leads to constitutive p110α activation in cells, and thus promotes oncogenic transformation, uncontrolled proliferation and metastasis, as well as drug resistance in breast cancer.^13^, ^14^ Beyond the direct antiproliferative effect on tumour cells, it has been reported that targeted PIK3CA^mut^ treatment can affect the microenvironment of breast cancer patients.^15^ Moreover, oncogenic gene mutation‐bearing tumours are associated with a highly suppressive TIME, which is represented by the infiltration of multiple types of suppressive immunocytes in multiple cancers.^16^, ^17^ Thus, clarifying the crosstalk between PIK3CA^mut^‐bearing tumours and TIME, and elucidating the vital molecular events involved in this process may help to develop more potent synergistic therapies and further improve the clinical efficacy for refractory advanced LBC patients.

Myeloid‐derived suppressor cells (MDSCs) are arguably the most important modulators of TIME and are the cornerstone of the immunosuppressive shield that protects the tumour from the host immune system and immunotherapy.^18^ Based on surface markers, three subtypes of MDSCs, including mononuclear myeloid‐derived suppressor cells (M‐MDSCs), polymorphonuclear MDSCs (PMN‐MDSCs) and early‐stage MDSCs (eMDSCs), have been described in mice and humans, among which eMDSCs are newly defined MDSCs with an immature phenotype and exceptional immunosuppressive ability.^19^, ^20^ Previously, we found that eMDSCs accumulated in breast cancer tissues and acted as predominant immunosuppressive cells in TIME, which predicted poor prognosis of LBC patients by inducing immune evasion and promoting tumour EMT.^21^, ^22^ Furthermore, it has been reported that constitutively activated PIK3CA and deleted TP53 recruit a drastically increased ratio of PMN‐MDSCs in head and neck cancers.^23^ Therefore, MDSCs may be a crucial coordinator to orchestrate the crosstalk between PIK3CA^mut^ tumours and TIME in LBC, although the concrete mechanisms remain to be elucidated.

In this investigation, we employed single‐cell mass cytometry (CyTOF), which merges flow cytometry with mass spectrometry,^24^ to describe the landscape of TIME in PIK3CA^mut^ LBC at single‐cell resolution and thus warrants a deeper understanding of the intricate characteristics of TIME and its spatial‐temporal association with LBC cells.^25^ CyTOF analysis revealed that PIK3CA^mut^ LBC exhibit immunosuppressive features of high MDSCs and low cytotoxic T‐cell infiltration in situ. We demonstrated that PIK3CA^mut^ activates the downstream Akt/STAT3 signalling pathway in LBC cells, which in turn directly increases the transcription of 5‐LOX and the production of LTB4, binding to BLT2 on eMDSCs and promoting their migration and infiltration into TIME. The growth of PIK3CA^mut^ tumours can be suppressed by blocking the PI3K/5‐LOX/LTB4 axis. Furthermore, combination therapy with a PI3K inhibitor and LTB4 antagonist significantly improved the antitumour efficacy of the anti‐PD‐1 antibody, as both the PI3K inhibitor and LTB4 antagonist reversed the excluded TIME (cold tumours) into the inflamed TIME (hot tumours) with increased expression of PD‐L1 and highly activated CD8^+^ T cells.^6^, ^26^ Therefore, the PI3K/5‐LOX/LTB4 axis not only clarified a distinct interactive pattern in LBC‐TIME crosstalk, but also sheds light on the promising future of synergistic treatment regimens of targeted therapy and immunotherapy for refractory advanced LBC patients.

## 2 MATERIALS AND METHODS

### 2.1 Patient information

The breast cancer patients included in our study were sourced from Tianjin Medical University Cancer Institute and Hospital (*n* = 62, Table S1), which contains 37 LBC patients (TJMU cohort; PIK3CA wild type, PIK3CA^wt^, 23 patients, PIK3CA^mut^, 14 patients). Lumpectomy was administered to the patients at the Breast Cancer Department. Prior therapeutic interventions such as chemotherapy or radiotherapy were not undertaken before performing lumpectomy. Furthermore, a total of 140 primary tissue samples derived from breast cancer were collected from a tissue microarray (TMA) acquired from Shanghai Xinchao (Table S2). These samples underwent surgical resection between August 2004 and December 2008. Additionally, this research study included the enrolment of 62 patients diagnosed with LBC, constituting the BRCA TMA cohort. The ethical committee of Tianjin Medical University Cancer Institute and Hospital granted approval for all tissue sample experiments, and patients provided written informed consent (Ek2021143).

### 2.2 Cell lines and cell culture

The human LBC cell lines T‐47D (Cellcook) and MCF‐7 (Cellcook) were cultured in RPMI‐1640 (GIBCO, C11875500BT) and DMEM (GIBCO, 12100046) supplemented with 10% FBS, respectively. The human breast epithelial cell line MCF‐10A (Cellcook) was maintained with endothelial cell medium (Pricella). The mouse mammary cancer cell line EO771 (American Type Culture Collection) was cultured in DMEM. PIK3CA^wt^, PIK3CA^E545K^ and PIK3CA^H1047R^ EO771 cell lines were established using the CRISPER/Cas9 system. The human embryonic kidney cell line HEK293T (Cellcook) was cultured in DMEM. Cells were cultured in conditions that imitated humanized environments, with a temperature of 37°C and a CO_2_ concentration of 5%. Furthermore, authentication through short tandem repeat (STR) genotyping was conducted to verify the cells.

### 2.3 Antibodies and reagents

Table S3 lists the antibodies and reagents employed in this study for various techniques, including immunoblotting (IB), immunohistochemistry (IHC), multiplex immunohistochemistry (mIHC), immunofluorescence (IF) and chromatin immunoprecipitation (ChIP) assays.

### 2.4 Bioinformatics analysis

We searched the TCGA database and collected 1083 cases of breast cancer patients with intact pathological and sequencing data. Only luminal A and luminal B molecular subtype cases of breast cancer, which contained a total of 696 cases, were included in the analysis (TCGA cohort; PIK3CA^wt^, 395 patients; PIK3CA^mut^, 301 patients). Additionally, RNA sequencing was performed on 37 breast cancer tissues from Tianjin Medical University Cancer Institute and Hospital (TJMU cohort). Differentially expressed genes between the PIK3CA^wt^ and PIK3CA^mut^ groups were analyzed using the DESeq R package (1.8.3). To further interpret these data, bioinformatics pathway enrichment analysis (http://www.bioinformatics.com.cn/) was performed to identify signalling pathways and biological processes affected by PIK3CA^mut^. Additionally, to identify gene expression in GSE2034 and GSE48408, we employed GEO2R. Motif position weight matrices and the binding sites between STAT3 and ALOX5 were predicted using the JASPAR website (http://jaspar.genereg.net/).

### 2.5 Mass cytometry

Mass cytometry (CyTOF) analysis was performed as described previously.^27^ The CyTOF staining panels are described in detail in Table S4. The tumours were digested and filtered using 40‐μm filters to isolate individual cells. To ensure the removal of erythrocytes, hypotonic lysis was performed on all isolated single cells at room temperature. To determine cell viability, a single wash with PBS was performed on cells, followed by a 2‐min incubation with Cell‐ID Cisplatin (Novogene) at a concentration of 2.5 μM. Subsequently, the cells underwent another wash with DPBS and were fixed in 1.6% paraformaldehyde (diluted in DPBS) for 2 min at room temperature. FlowJo software was used to determine the percentages of each cell population.

### 2.6 Immunohistochemistry

To conduct IHC staining, the LBC tissues or TMA that were embedded in paraffin needed to undergo deparaffinization and rehydration. H_2_O_2_ was used to prevent interference from endogenous peroxidase. Then, the cells were incubated overnight with primary antibodies and subsequently treated with biotinylated secondary antibodies utilizing the immunoperoxidase method based on the avidin–biotin complex (Table S3). Finally, ImageScope software from Leica Biosystems was employed to visualize and analyze all the specimens. According to the number of positive cells, the median number of MDSCs was used as the grouping method. The staining score was calculated based on the intensity × proportion of stained tumour cells, in which less than six and ≥six were defined as low and high 5‐LOX expressions, respectively.

### 2.7 Multiplex immunohistochemistry

mIHC is an IF‐based multiplex hybridization method that efficiently and rapidly stains multiple markers simultaneously using the Opal Manual kit (Opal 7 staining kit, Akoya Biosciences) according to the manufacturer's protocol.^28^ Following the application of heat treatment to the tissues, the staining procedure entails the exclusion of the primary and secondary antibodies, while preserving the fluorescence molecules coupled with TSA. Following several rounds of staining, the application of the slides occurs for whole‐slide imaging using the Mantra quantitative pathology imaging system.

### 2.8 Immunofluorescence staining

EO771 PIK3CA^wt^ or PIK3CA^mut^ cells were seeded onto 18‐mm coverslips, stabilized overnight at 37°C. In brief, blocking was performed with 5% bovine serum albumin, followed by incubation with primary antibodies and secondary antibodies with fluorescent labels. The nuclei were stained using DAPI (Thermo). LSM 880 laser scanning (Zeiss) was employed to visualize the samples.

### 2.9 Identification of the prevalence of PIK3CA mutations in tissues

To detect PIK3CA mutations within the tissue sections, fluorescence in situ hybridization (FISH) was utilized. First, the sections underwent deparaffinization using xylene, followed by dehydration in ethanol and subsequent air drying. Next, the sections were subjected to treatment with 2× sodium chloride‐sodium citrate (SSC). Subsequently, they were rinsed in 2× SSC at room temperature for a period of 5 min, after which they underwent digestion using .25 mg/mL Protease K in 2× SSC for 20 min. The slides were then rinsed once again in 2× SSC, dehydrated using a series of ethanol solutions, and thoroughly air‐dried. After digestion with restriction endonuclease, single‐stranded target DNA sequences were exposed by lambda exonuclease. The padlock probe was hybridized and cyclized with T4 DNA ligase. To generate adequate quantities of DNA for the synthesis of FISH probes, rolling circle amplification (RCA) was employed to amplify the DNA. The padlock sequences are listed in Table S5.

### 2.10 Protein extraction and immunoblotting

After performing the proposed experiments, cell lysis was carried out using RIPA buffer. Subsequently, proteins were separated on gels using SDS‐PAGE, and subsequently transferred to PVDF membranes. The membranes were sequentially incubated with primary antibodies and HRP‐conjugated secondary antibodies, and enhanced chemiluminescence reagent (Thermo) was used to observe the protein bands.

### 2.11 Quantitative real‐time PCR

TRIzol reagent was applied to extract total RNA (Invitrogen). Then, the samples were transcribed into cDNA using PrimerScriptTM RT Master Mix (TaKaRa). A Roche LightCycler 480 II real‐time PCR system was employed for quantitative real‐time PCR. To normalize the expression levels of target genes, GAPDH was utilized as a loading control (2^−ΔΔCt^ method). The primers are listed in Table S6.

### 2.12 Chromatin immunoprecipitation assay

The ChIP experiment was conducted utilizing the ChIP assay kit (Cell Signaling Technology, #9003) to evaluate the interactions between STAT3 and the promoter of ALOX5 in accordance with the protocol. The primers are listed in Table S7.

### 2.13 Dual‐luciferase reporter assay

pGL3‐promoter vectors and HEK293T cells were chosen, and the dual‐luciferase reporter assay system (Promega) was used according to the manufacturer's instructions.

### 2.14 Live cell imaging

MDSCs were cocultured with PIK3CA^wt^, PIK3CA^E545K^ or PIK3CA^H1047R^ EO771 cells. The cell mixture was then transferred to a 96‐well culture plate containing a glass bottom dish. Live cell imaging was performed for 24 h to capture the interactions between MDSCs and cancer cells. To obtain quantitative data, two independent professionals quantified the interactions using Leica CTR6000 microscopy at 20× magnification.

### 2.15 Transwell chemotaxis

An 8‐μm pore transwell chamber (Corning) was used for chemotaxis. MDSCs were placed on the top, and PIK3CA^wt^, PIK3CA^E545K^ or PIK3CA^H1047R^ EO771 cells were planted in the bottom chamber. Cells were stained and counted after culturing.

### 2.16 Cell isolation

T cells were purified from the spleens of the mice (>95% purity), and activated with CD3/28 beads. MDSCs were isolated from the tumours (>95% purity). Bone marrow cells were stimulated with IL‐6 (20 ng/mL, murine IL‐6, PeproTech) and GM‐CSF (40 ng/mL, murine GM‐CSF, PeproTech) for 72 h to induce MDSCs.

### 2.17 Flow cytometry

Flow cytometry assay was conducted as previously reported.^29^ Single‐cell suspensions were collected and stained with monoclonal antibodies (1 μg/10^6^ cells/100 μL). Table S8 contains the antibodies employed in the assay.

### 2.18 T‐cell suppression assay

A T‐cell suppression assay was performed, as previously described.^27^ MDSCs and CFSE‐labelled T cells were placed at a ratio of 0:1 or 1:1. After 72 h, the CFSE (Invitrogen) intensity was measured using a FACS Canto II apparatus by identifying the peaks. These peaks denoted the division times of the cells, with division times zero to two classified as low proliferation and division times three to four as high proliferation.

### 2.19 Metabolite analysis targeting arachidonic acid signalling

Shanghai Bioprofile Biotechnology Co., Ltd conducted the analysis of arachidonic acid (AA) metabolites. The samples were collected, centrifuged and homogenized on ice. Then, the samples were dried and dissolved. The results were analyzed using ACQUITY ultra performance liquid chromatography (Vanquish, UPLC, Thermo) in conjunction with a mass spectrometer (Q Exactive, Thermo).

### 2.20 Enzyme‐linked immunosorbent assay

AA, 5‐HETE, 12‐HETE, 15‐HETE, LTB4, LTC4, LTD4 and IFN‐γ levels (Table S3) were evaluated utilizing enzyme‐linked immunosorbent assay (ELISA) kits according to the manufacturer's guidelines.

### 2.21 Animal experiments

The Animal Care and Use Committee of Tianjin Medical University Cancer Institute and Hospital has granted approval to all animal protocols (AE2021016). A total of 5 × 10^5^ EO771^wt^, EO771^E545K^ or EO771^H1047R^ cells were injected subcutaneously into a 6‐week‐old female C57 mice until xenografts were established (>100 mm^3^), which were sacrificed to harvest tumours for further studies. To assess the influence of infiltrated MDSCs in situ on LBC tumour growth, the utilization of SOCS3 conditional knockout mouse models described previously was imperative.^29^ PIK3CA^mut^ EO771 xenograft‐bearing mice were administered saline, alpelisib (20 mg/kg), pembrolizumab (anti‐PD‐1, 10 mg/kg), LY255283 (20 mg/kg) or combined therapies every 3 days. Bioluminescence imaging systems were employed to assess the tumour size at intervals of 7 days using in vivo imaging. After 21 days, the animals were euthanized, and the weight and dimensions of the tumours were measured.

### 2.22 Statistical analysis

Statistical analyses were performed using GraphPad Prism 8. Two‐tailed Student's *t*‐tests were utilized to conduct the comparisons between the two groups. For multiple‐group comparisons, either one‐way or two‐way ANOVA was employed, followed by a Tukey correction to compare each group. The Pearson correlation test was performed to assess the correlation. Analysis of the survival data was performed using the log‐rank (Mantel–Cox) test. The mean ± standard deviation (SD) was used to present the data. A value of *p* < .05 was considered statistically significant.

## 3 RESULTS

### 3.1 LBC patients with PIK3CA^mut^ were featured with a suppressive TIME with aggravated MDSC infiltration

**FIGURE 1:**
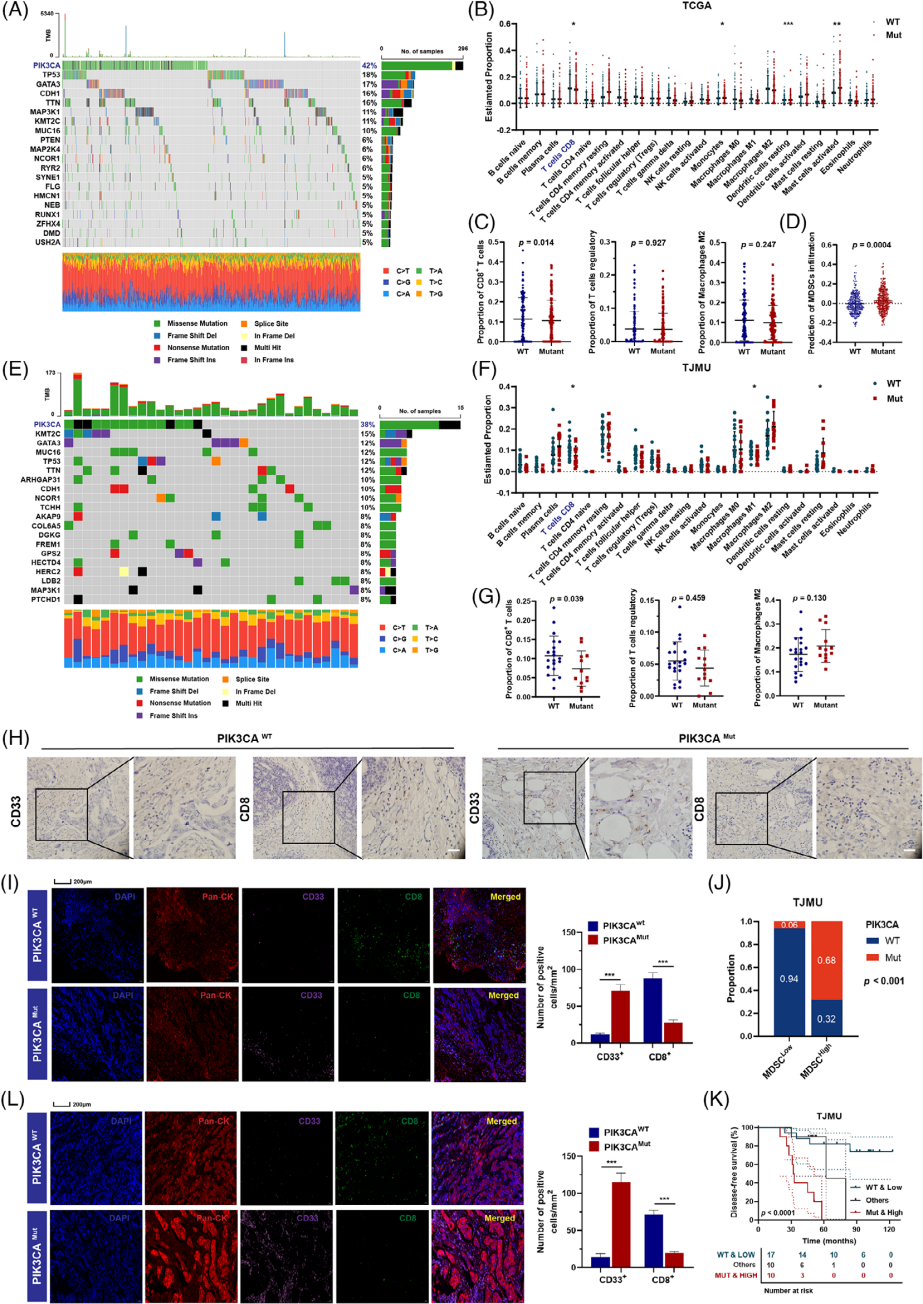
LBC patients with PIK3CA^mut^ were featured with a suppressive TIME with aggravated MDSC infiltration. (A) Waterfall diagram indicating the overall number of mutations for the most commonly mutated genes and their prevalence in the TCGA cohort (*n* = 696). (B) The TCGA cohort's immune infiltration status was analyzed by CIBERSORT, and the frequency of CD8^+^ T cells was higher in PIK3CA^wt^ tumours. (C) The proportions of M2 macrophages, Tregs, CD8^+^ T cells and MDSCs (D) in the TCGA cohort. (E) Waterfall diagram showing the overall number of mutations in genes in the TJMU cohort (*n* = 37). (F and G) The estimated proportion of immune cells in the TJMU cohort was analyzed by CIBERSORT. (H) Images of immunohistochemical staining to assess the infiltration of T cells (CD8^+^) and MDSCs (CD33^+^) in LBC. Scale bar: 100 μm. (I) mIHC staining and quantification of MDSCs, T cells and tumour cells in the TJMU cohort (*n* = 37). (J) High infiltration of CD33^+^ MDSC was positively correlated with PIK3CA^mut^ in the TJMU cohort. (K) High infiltration of MDSCs plus PIK3CA^mut^ predicted shorter survival in LBC. (L) mIHC staining results for MDSCs, T cells and tumour cells in the BRCA TMA cohort (*n* = 62). Data represent mean ± SD. *p < .05, **p < .01, ***p < .001.

PIK3CA is one of the most frequently mutated genes in breast cancer. Somatic mutant profiles of the TCGA cohort were visualized by waterfall plots, which showed that PIK3CA was the most frequently mutated gene (Figure 1A). To conduct a comprehensive analysis regarding the variance in immune cell infiltration between the PIK3CA^wt^ and PIK3CA^mut^ groups, we employed the CIBERSORT algorithm to examine alterations in the composition of immune cell subgroups within the TCGA cohort. The results revealed that PIK3CA^mut^ tumours have a significantly low infiltration of cytotoxic T cells (Figure 1B). The results revealed a statistically significant change in CD8^+^ T cells rather than Tregs and M2 macrophages in the TIME of PIK3CA^mut^ LBC samples (Figure 1C). Furthermore, using TIMER2.0, we observed higher MDSC infiltration in PIK3CA^mut^ LBC samples in the TCGA cohort (Figure 1D). Based on the median number of MDSCs, we stratified the LBC patients into two groups (TJMU cohort, median = 4.7; BRCA TMA cohort, median = 5.0). The above results were verified in the TJMU cohort (Figure 1E). PIK3CA was the most commonly mutated gene in LBC in both the TCGA cohort and the TJMU cohort. CIBERSORT analysis demonstrated significantly lower infiltration of CD8^+^ T cells (*p* < .05) in PIK3CA^mut^ LBC samples (Figure 1F,G). To establish the significance of PIK3CA^mut^ in promoting TIME suppression, we conducted an IHC assay. The IHC analysis of PIK3CA^mut^ tumours provided additional evidence supporting the absence of T‐cell infiltration and the notable presence of MDSCs (Figure 1H), as opposed to Tregs and M2 macrophages (Figure S1A). The statistical analysis is shown in Figure S1B. Consistently, mIHC analysis revealed a lack of CD8^+^ T cells and infiltration of MDSCs in the PIK3CA^mut^ LBC TIME in the TJMU cohort (Figure 1I). Furthermore, we examined the correlation between MDSC infiltration and PIK3CA^mut^, and found that the number of CD33^+^ MDSCs in situ was positively correlated with PIK3CA^mut^ in tumours (Figure 1J). Interestingly, further stratification of patient groups based on high MDSC infiltration plus PIK3CA^mut^ predicted shorter disease‐free survival in LBC (Figure 1K). The same results were obtained in another cohort, the BRCA TMA cohort. According to the RCA‐FISH results, patients were separated into the PIK3CA^wt^ group and PIK3CA^mut^ group in the BRCA TMA cohort (Figure S1C). Similarly, mIHC analysis revealed a lack of CD8^+^ T cells and high infiltration of MDSCs in the PIK3CA^mut^ TIME (Figure 1L). A positive correlation was detected between MDSC recruitment in tissues and PIK3CA^mut^ status (Figure S1D), and PIK3CA^mut^ patients with high MDSC infiltration suffered from the worst clinical outcome and shortest overall survival (Figure S1E). MDSCs, a heterogeneous population of immature myeloid cells, have rather diverse phenotypes in cancer. To further validate our conclusions, we used another current consensus marker, CD84, for the characterization of MDSC subsets (Figure S1F). This *t*‐test result shows a high degree of relative agreement (Figure S1G). These data indicated that PIK3CA^mut^ correlated with high MDSCs and low T‐cell infiltration in primary LBC tissues.

### 3.2 PIK3CA^mut^ promoted an immunosuppressive TIME in tumour tissues of LBC xenograft‐bearing mice

**FIGURE 2:**
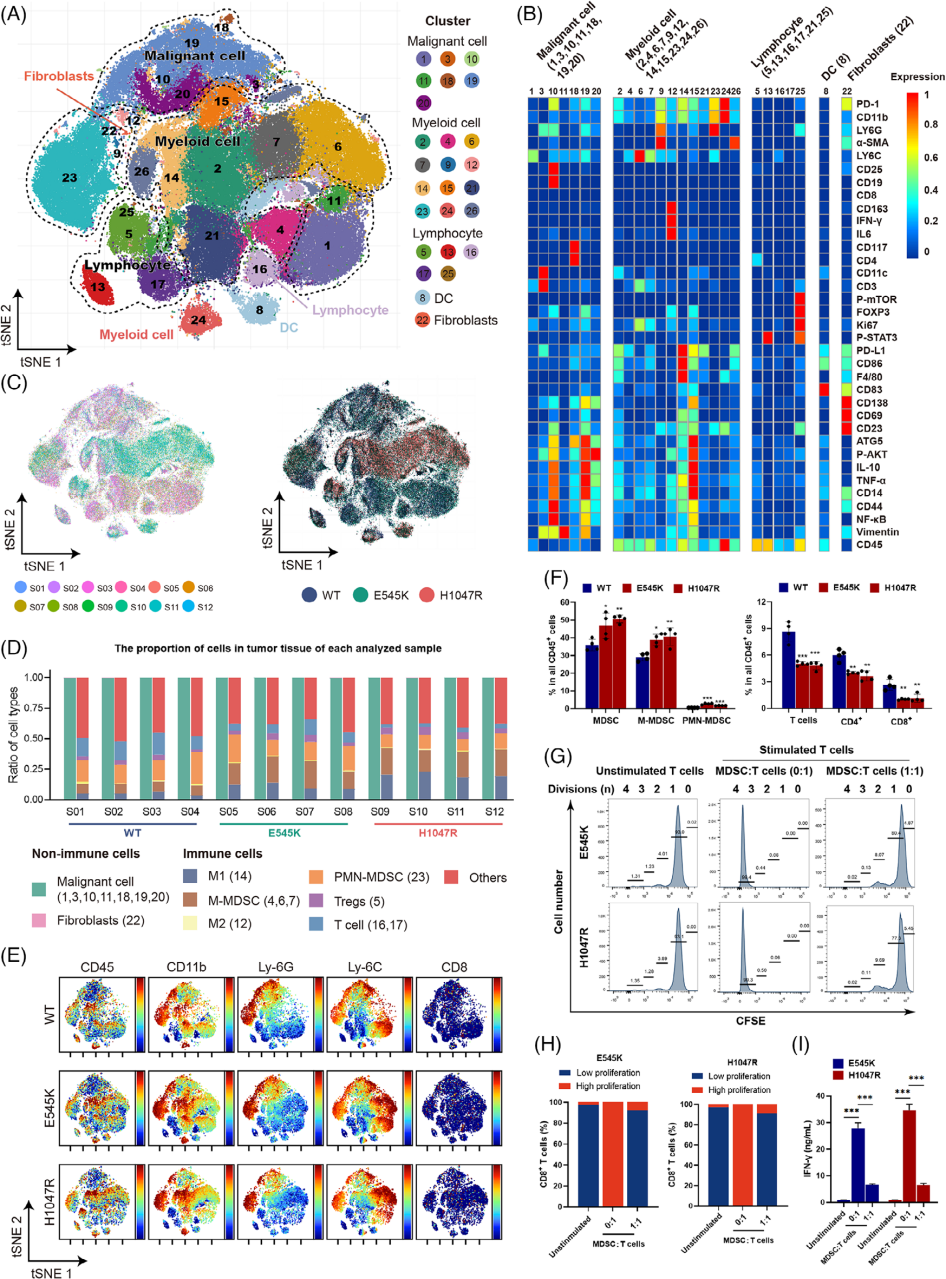
PIK3CA^mut^ promoted an immunosuppressive TIME in tumour tissues of LBC xenograft‐bearing mice. (A) t‐SNE analysis of cells from PIK3CA^mut^ tumours coloured by relative expression of CyTOF markers. (B) Heatmap showing the expression of marker proteins in the indicated cell types. The top bars label the clusters corresponding to the specific cell types. The number in the brackets corresponds to the cluster number in (A). (C) The t‐SNE plot, showing the cell origins by colour, sample origin (left panel) and PIK3CA^mut^ or PIK3CA^wt^ origin (right panel). (D) Histogram indicating the proportion of cells in the tumour tissues of each sample analyzed. (E) t‐SNE analysis of immune cells from PIK3CA^mut^ tumours and the populations are indicated. (F) Quantification of tumour‐infiltrating cells in PIK3CA^mut^ tumours by flow cytometry. (G) Representative CFSE flow cytometry histograms showing the effect of MDSCs isolated from PIK3CA^mut^ tumours on T‐cell proliferation in vitro and the summarized results (H). (I) IFN‐γ secretion by T cells was measured by ELISA. Data represent mean ± SD. *p < .05, **p < .01, ***p < .001.

To investigate the immunomodulatory effects of PIK3CA^mut^ on the TIME in vivo, we utilized a mouse luminal mammary cancer model. Single‐cell CyTOF was performed to observe detailed characteristics of TIME in PIK3CA^mut^ tumours. We compared the amount and distribution of multiple cell subtypes, and found that 26 subtypes with different lineages were detected in xenografts (Figure 2A). The heatmap shows the levels of proteins in each subtype, which can be divided into five groups, including malignant cells, myeloid cells, lymphocytes, DCs and fibroblasts (Figure 2B). Cytobank‐based viSNE analysis of CyTOF data revealed a complex cellular landscape of cancer cells, immunocytes and other cells in which significant disparity in the distribution of myeloid lineage subtypes was detected among 12 xenografts harbouring different genetic features on the PIK3CA gene, including ‘wild type (wt)’, ‘E545K’ and ‘H1047R’ (Figure 2C). It is worth mentioning that the major cell population consisted of infiltrating myeloid cells (CD45^+^CD11b^+^), which increased in xenografts harbouring the E545K or H1047R mutation (Figure 2D). Meanwhile, MDSCs, specifically M‐MDSCs dramatically increased, and T cells significantly decreased in PIK3CA^mut^ tumours (Figure 2E). FlowJo analysis confirmed lower proportions of T cells and higher proportions of MDSCs in both the PIK3CA^E545K^ and PIK3CA^H1047R^ groups than in the PIK3CA^wt^ group (Figure 2F). The results revealed a significant decrease in the percentage of CD8^+^ T cells in PIK3CA^mut^ tumours. CFSE peaks indicate the division times, and 0−2 and 3−4 represent low and high proliferations, respectively. These CD45^+^CD11b^+^ MDSCs strongly suppressed anti‐CD3/CD28 antibody‐induced T‐cell proliferation (Figure 2G,H), and inhibited IFN‐γ production by T cells (Figure 2I). We concluded that PIK3CA^mut^ promoted a suppressive TIME in LBC tissues by recruiting MDSCs and excluding cytotoxic T cells.

### 3.3 AA metabolism reprogramming was detected in PIK3CA^mut^ LBC by activating the 5‐LOX pathway

**FIGURE 3:**
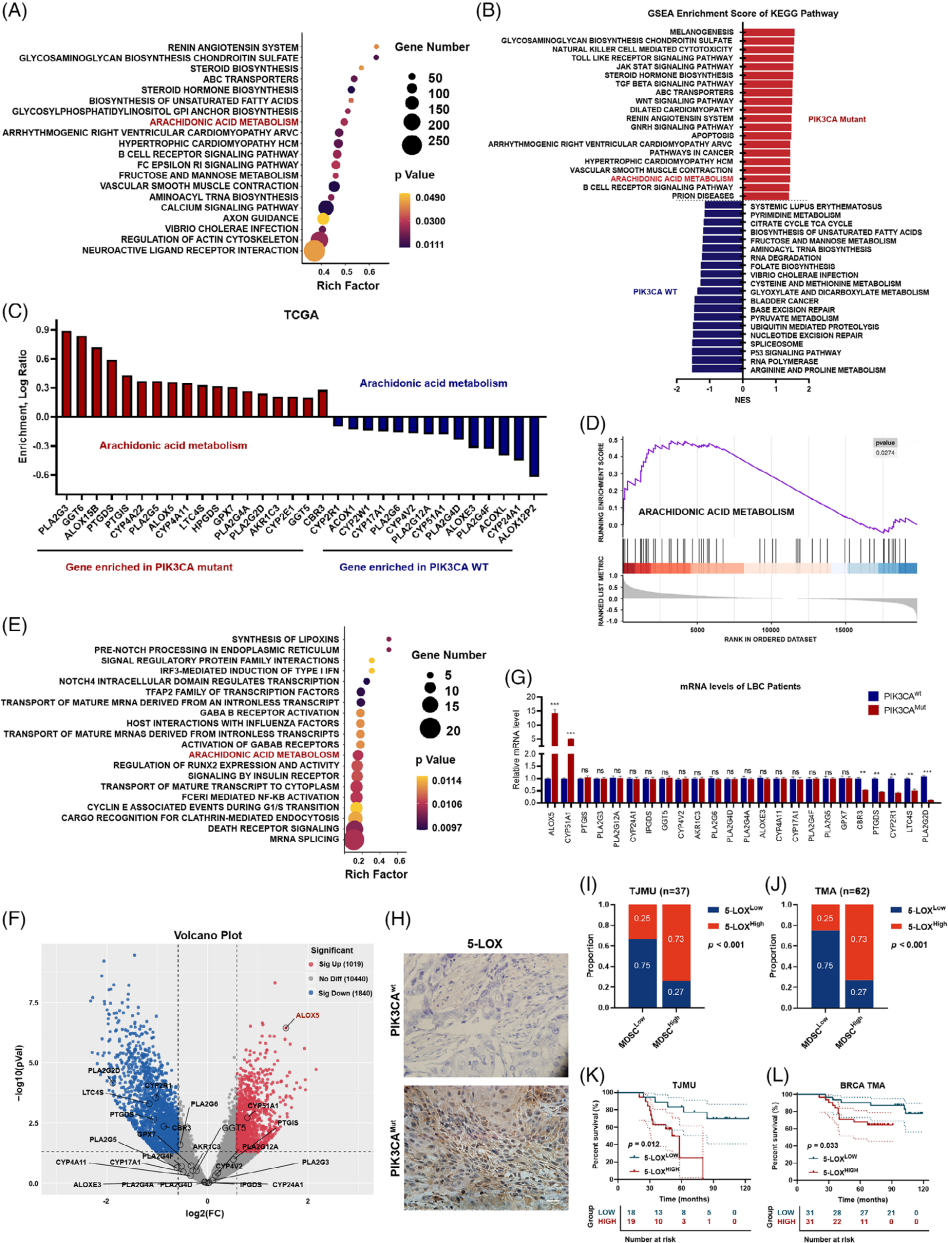
AA metabolism reprogramming was detected in PIK3CA^mut^ LBC by activating the 5‐LOX pathway. (A) Enriched KEGG terms of genes positively co‐expressed with PIK3CA^mut^ in the TCGA cohort. (B) Gene‐set enrichment analysis of enriched KEGG pathways. (C) Log_2_ fold change of representative enriched genes in PIK3CA^mut^ tumours. (D) GSEA enrichment plot for AA metabolism pathways (E) Enriched KEGG terms of genes positively co‐expressed with PIK3CA^mut^ in TJMU cohort. (F) Volcano plot of the overexpressed genes in PIK3CA^mut^ in the TJMU cohort. (G) The mRNA levels of 24 genes in TJMU cohort. (H) Images of immunohistochemical staining to assess the level of 5‐LOX in PIK3CA^wt^ and PIK3CA^Mut^ tissues. Scale bar: 50 μm. (I) The overexpression of 5‐LOX was positively correlated with high infiltration of MDSCs in the TJMU cohort and in the BRCA TMA cohort (J). (K and L) Kaplan–Meier survival curve analysis showed that 5‐LOX overexpression indicated poor prognosis in LBC. Data represent mean ± SD. *p < .05, **p < .01, ***p < .001.

To elucidate the downstream mechanisms by which PIK3CA^mut^ promotes an immunosuppressive profile in TIME in LBC, we conducted KEGG pathway enrichment and GSEA analyses in the TCGA cohort. We focused on metabolism‐related pathways and found some lipid metabolism‐related pathways, including AA metabolism, which was enriched in PIK3CA^mut^ tumours (Figure 3A). We used Venn diagrams to take the intersection of upregulated pathways related to PIK3CA^mut^ tumours in the TCGA cohort, TJMU cohort and MDSC infiltration‐related pathways and obtained 33 targets (Figure S2A, Table S9). Among the enriched pathways, we found that AA metabolism was upregulated in PIK3CA^mut^ tumours (Figure 3B). Furthermore, we detected AA metabolism‐related genes and found that the mRNA levels of 18 key enzyme‐encoding genes, such as PLA2G3, GGT6, ALOX15B, PTGDS, PTGIS, PLA2G5, ALOX5, LTC4S, PLA2G4A and PLA2G2D, increased notably (Figure 3C). Similar results were obtained from the gene set enrichment analysis (GSEA) (Figure 3D). However, further analysis of GSE216871 revealed that some of the genes were not phase variably expressed, such as PLA2G3 and LTC4S (Figure S2B). Then, we used RNA‐seq from the TJMU cohort to validate the above results and confirmed that AA metabolism was among the top enriched pathways in PIK3CA^mut^ samples (Figure 3E). We compared the upregulated genes between the TCGA cohort and the TJMU cohort in AA metabolism and found that ALOX5 was included in both cohorts (Figure 3F). qPCR assays were performed to validate the expression of 24 genes, as shown in Figure 3F, and found that ALOX5 was the most upregulated gene in PIK3CA^mut^ samples (Figure 3G). Furthermore, IHC verified the increased expression of 5‐LOX in PIK3CA^mut^ patients (Figure 3H). Thus, the ALOX5 gene was identified as the key coordinator along the AA pathway in LBC. Further analysis targeting the AA pathway detected higher levels of 5‐LOX (ALOX5‐encoded protease) than other key enzymes in the AA metabolism pathway, such as 12‐LOX, 15‐LOX and COX2, in the PIK3CA^mut^ groups (Figure S2C,D). ALOX5 is a key gene in the LOX pathway of AA metabolism, which encodes the key enzyme of 5‐LOX. To assess the predominance of 5‐LOX transcription in MDSC recruitment, we focused on the relationship between 5‐LOX and MDSC infiltration. The infiltrated MDSCs were positively correlated with 5‐LOX expression in both the TJMU cohort (Figure 3I) and BRCA TMA cohort (Figure 3J). We also confirmed the results in public breast cancer datasets according to an established MDSC signature we reported previously^22^ (Figure S2E–G). Moreover, elevated 5‐LOX expression was associated with poor prognosis in LBC patients in these two cohorts (Figure 3K,L) and the public database (Figure S2H). Thus, the ALOX5 gene was identified as the key downstream molecule.

### 3.4 PIK3CA^mut^ initiated ALOX5‐associated AA metabolism reprogramming by activating the STAT3 signalling pathway

**FIGURE 4:**
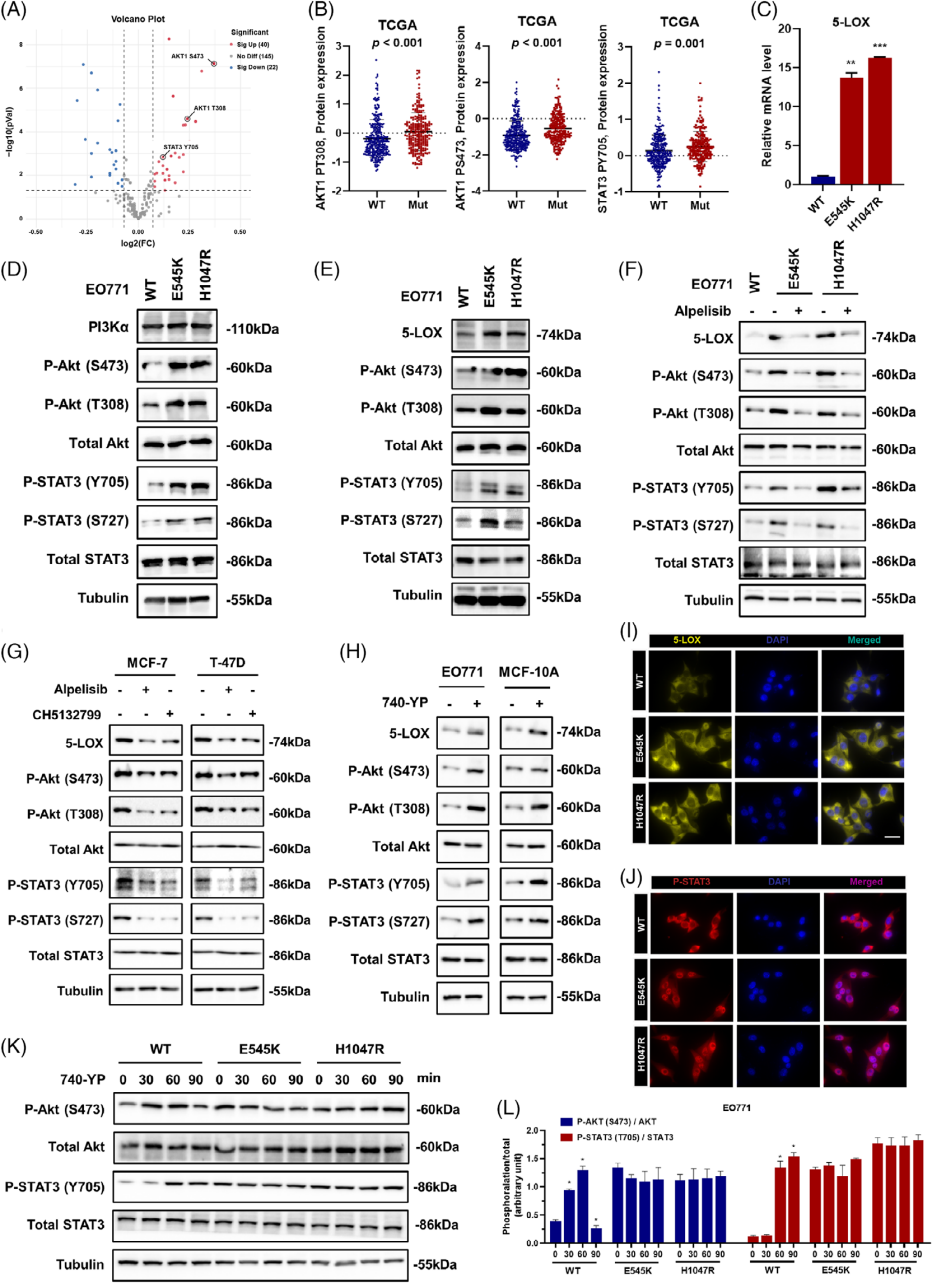
PIK3CA^mut^ initiated ALOX5‐associated AA metabolism reprogramming by activating the STAT3 signalling pathway. (A) Differential gene volcano map. (B) The levels of P‐Akt (T308), P‐Akt (S473) and P‐STAT3 (Y705) in TCGA database in LBC. (C) The mRNA level of 5‐LOX was analyzed by qPCR assay in EO771^wt^, EO771^E545K^ and EO771^H1047R^ cells. (D) The protein expression of PI3Kα, P‐Akt (S473), P‐Akt (T308), P‐STAT3 (Y705) and P‐STAT3 (S727) was detected in EO771^wt^, EO771^E545K^ and EO771^H1047R^ cells. (E) The protein expression of 5‐LOX, P‐Akt (S473), P‐Akt (T308), P‐STAT3 (Y705) and P‐STAT3 (S727) was detected in EO771^wt^, EO771^E545K^ and EO771^H1047R^ cells. (F) The protein expression of 5‐LOX, P‐Akt (S473), P‐Akt (T308), P‐STAT3 (Y705) and P‐STAT3 (S727) was detected in EO771^wt^, EO771^E545K^ and EO771^H1047R^ cells treated with alpelisib. (G) The protein expression of 5‐LOX, P‐Akt (S473), P‐Akt (T308), P‐STAT3 (Y705) and P‐STAT3 (S727) was detected in MCF‐7 and T‐47D cells treated with alpelisib or CH5132799. (H) The protein expression of 5‐LOX, P‐Akt (S473), P‐Akt (T308), P‐STAT3 (Y705) and P‐STAT3 (S727) was detected in EO771 and MCF‐10A cells treated with 740‐YP. (I) Representative images of immunofluorescence staining of 5‐LOX and P‐STAT3 (J) in EO771^wt^, EO771^E545K^ and EO771^H1047R^ cells. Scale bar: 20 μm. (K and L) The protein expression of P‐Akt (S473) and P‐STAT3 (Y705) was detected in EO771^wt^, EO771^E545K^ and EO771^H1047R^ cells treated with 740‐YP. Data represent mean ± SD. *p < .05, **p < .01, ***p < .001.

To explore the mechanism involved in PIK3CA^mut^‐associated upregulation of ALOX5 in LBC cells, we screened the most significantly upregulated proteins in PIK3CA^mut^ cancer cells using proteomics data from the TCGA database, and found increased phosphorylation levels of both Akt and STAT3 proteins in PIK3CA^mut^ patients in the TCGA cohort (Figure 4A,B). As a previous study indicated that STAT3 could bind to the promoter region of ALOX5 in cholangiocarcinoma,^30^ we started to determine whether PIK3CA^mut^ stimulated 5‐LOX expression by regulating the phosphorylation of Akt and STAT3 in vitro. We established PIK3CA^wt^, PIK3CA^E545K^ and PIK3CA^H1047R^ EO771 cell lines using the CRISPR/Cas9 system and found that the mRNA level of 5‐LOX was upregulated in PIK3CA^mut^ cells compared to PIK3CA^wt^ cells (Figure 4C). Interestingly, both the PI3K/Akt signalling pathway and STAT3 signalling pathway were activated in the PIK3CA^E545K^ and PIK3CA^H1047R^ EO771 cell lines (Figure 4D), and the statistical analysis is shown in Figure S3A. Consistently, Western blot analysis verified the increased expression of p‐Akt (both S473 and T308), p‐STAT3 (both Y705 and S727) and 5‐LOX in PIK3CA^mut^ cells (Figure 4E). The statistical analysis is shown in Figure S3B. Alpelisib, a potent and selective PI3Kα inhibitor, significantly inhibited the upregulation of p‐Akt (both S473 and T308), p‐STAT3 (both Y705 and S727) and 5‐LOX in PIK3CA^mut^ cells (Figure 4F). Statistical analysis is shown in Figure S3C. Furthermore, the results were validated using the human LBC cell lines T‐47D (PIK3CA^H1047R^) and MCF‐7 (PIK3CA^E545K^) using PI3K inhibitors (alpelisib and CH5132799), in which dramatic decreases in the levels of p‐Akt, p‐STAT3 and 5‐LOX were detected (Figure 4G). The statistical analysis is shown in Figure S3D. In contrast, we treated PIK3CA^wt^ EO771 cells and mammary epithelium cells MCF‐10A with 740‐YP, a PI3K activator, and found notable increases in p‐Akt (both S473 and T308), p‐STAT3 (both Y705 and S727) and 5‐LOX proteins (Figure 4H). The statistical analysis is shown in Figure S3E. In addition, the expression of 5‐LOX was evaluated (Figure 4I) in the cytoplasm of PIK3CA^mut^ cells, and the nuclear translocation of p‐STAT3 was elevated (Figure 4J) by immunofluorescence. E545K and H1047R are both activating mutations that trigger persistent activation signals along the PI3K pathway. Therefore, 740‐YP could not induce a remarked change in the ratio of phosphorylation/total in EO771^mut^ cells, as it did in EO771^wt^ cells (Figure 4K,L). Thus, we concluded that PIK3CA^mut^ aggravated 5‐LOX‐associated AA metabolism reprogramming by activating the STAT3 signalling pathway.

### 3.5 PIK3CA^mut^ activated the Akt/STAT3 axis to induce 5‐LOX transcription and recruit MDSC infiltration

**FIGURE 5:**
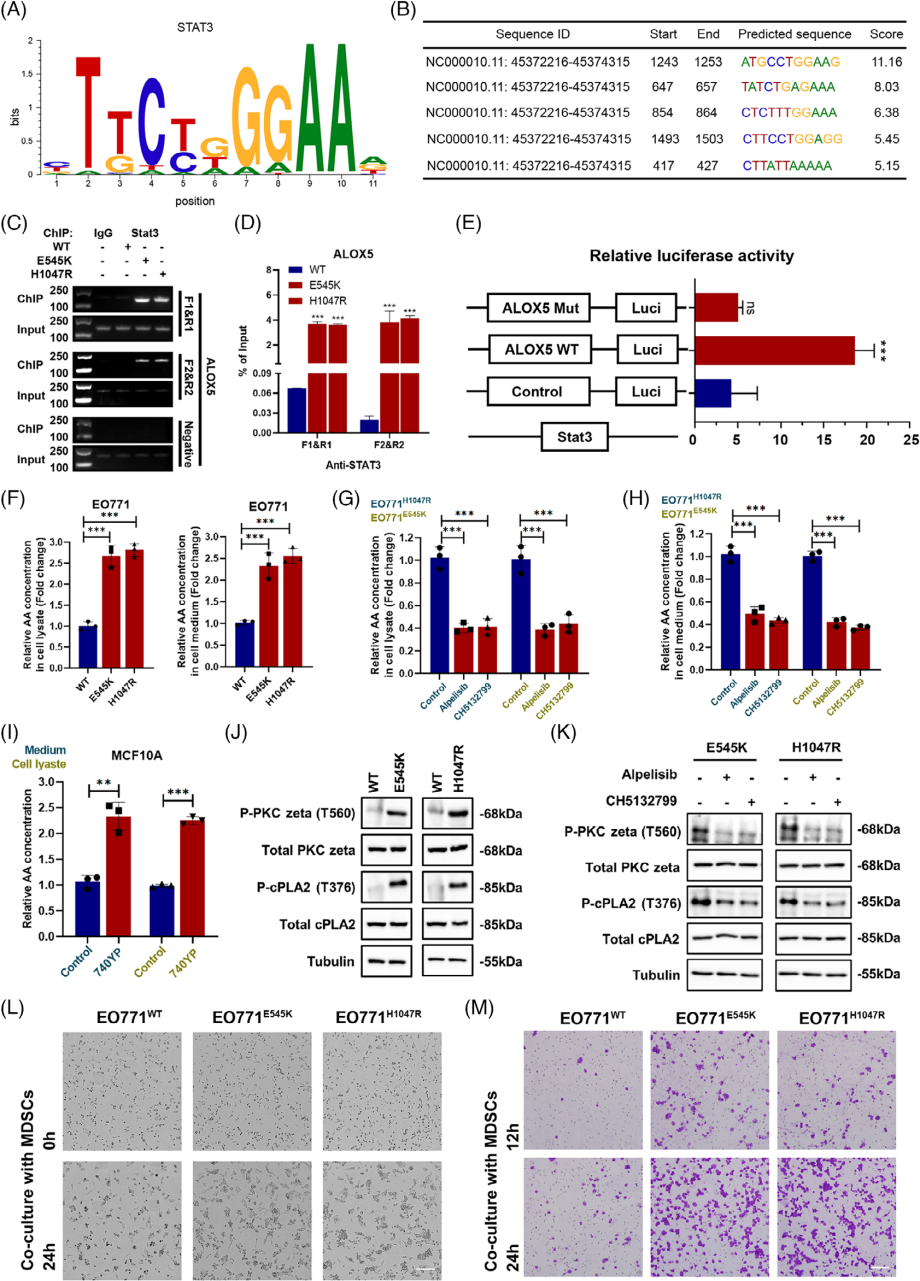
PIK3CA^mut^ activated the Akt/STAT3 axis to induce 5‐LOX transcription and recruit MDSC infiltration. (A) STAT3‐binding motif. (B) Prediction of STAT3‐binding sites within ALOX5 promoter region, JASPAR database. Representative images of conventional PCR (C) and quantitative PCR analysis (D) showing STAT3 binding to the ALOX5 promoter region. (E) The results of the dual‐luciferase reporter gene assay. (F) ELISA results of AA from EO771^WT^, EO771^E545K^ and EO771^H1047R^ cells lysate and medium. (G) ELISA results of AA from EO771^E545K^ and EO771^H1047R^ cell lysates treated with alpelisib or CH5132799. (H) ELISA results of AA from EO771^E545K^ and EO771^H1047R^ cell medium treated with alpelisib or CH5132799. (I) The ELISA results of AA from MCF‐10A cell lysate and medium. (J) The protein expression of P‐PKC zeta (T560) and P‐cPLA2 (T376) was detected in EO771^WT^, EO771^E545K^ and EO771^H1047R^ cells. (K) The protein expression of P‐PKC zeta (T560) and P‐cPLA2 (T376) was detected in EO771^WT^, EO771^E545K^ and EO771^H1047R^ cells treated with alpelisib or CH5132799. (L) MDSCs cocultured with PIK3CA^mut^ cells display more chemotactic activity in the live cell imaging assay and (M) the in vitro chemotaxis assay. Scale bar: 100 μm. Data represent mean ± SD. *p < .05, **p < .01, ***p < .001.

To define the regulatory effect of STAT3 on 5‐LOX transcription, we predicted the binding motif of the transcription factor STAT3 (Figure 5A). The predicted sequence motif, which STAT3 may bind to the promoter region of 5‐LOX, is shown in Figure 5B. The top two high‐scoring sequences were selected for in vitro binding assays, and ChIP assays revealed that the binding of STAT3 to the 5‐LOX promoter at either binding site was significantly enhanced by PIK3CA^mut^ (Figure 5C,D). Next, the dual luciferase reporter assay was performed to assess its transcriptional activity, which indicated that transfection of STAT3 plasmids resulted in the conspicuous upregulation of the luciferase activity of 5‐LOX^wt^ rather than 5‐LOX^mut^ reporter (Figure 5E). Furthermore, we applied siRNA to inhibit the expression of Akt or STAT3 in PIK3CA^mut^ cells. ELISA showed that the level of AA was lower in cell supernatants in si‐STAT3‐treated cells (Figure S3F), and the Western blot analysis revealed that the expression of 5‐LOX was significantly decreased in si‐Akt‐treated (Figure S3G) and si‐STAT3‐treated cells (Figure S3H). These results demonstrate that PIK3CA^mut^ activated the Akt/STAT3 axis to induce the transcription and expression of the 5‐LOX gene. Then, we compared AA levels in cell lysates and supernatants, which are precursors of HETEs and leukotrienes. ELISA showed that PIK3CA^mut^ cells produced significantly more AA in cell lysates and supernatants than PIK3CA^wt^ cells (Figure 5F). The results were validated in the human LBC cell lines T‐47D (PIK3CA^H1047R^) and MCF‐7 (PIK3CA^E545K^) pretreated with PI3K inhibitors, in which a significant decrease in the levels of AA was detected in the cell lysate (Figure 5G) and cell medium (Figure 5H). Conversely, PI3K activator‐treated MCF‐10A cells showed a significant increase in the levels of AA (Figure 5I). Western blot analysis verified the increased expression of p‐PKCζ (T560) and p‐cPLA2 (T376) in PIK3CA^mut^ cells (Figure 5J); importantly, this effect was greatly reversed by PI3K inhibitors (Figure 5K). To assess the predominance of PIK3CA^mut^ in MDSC recruitment, we used a chemotaxis assay composed of either PIK3CA^wt^ or PIK3CA^mut^ cells and MDSCs in vitro. The results of live cell imaging showed that the cell–cell contacts of cancer cells and MDSCs in the PIK3CA^mut^ group were significantly increased compared to those in the PIK3CA^wt^ group (Figure 5L). We further performed transwell coculture in vitro and found that MDSCs cocultured with PIK3CA^mut^ cells displayed more chemotactic activity in vitro (Figure 5M). Furthermore, we applied siRNAs to inhibit Akt, STAT3 and 5‐LOX enzyme expression in PIK3CA^mut^ cells. The results of the transwell chemotaxis assay in vitro showed that the recruitment of MDSCs can be significantly reduced in the presence of inhibition of the Akt/STAT3 axis and 5‐LOX (Figure S3I). Together, these data reinforced that the Akt/STAT3 axis was activated in PIK3CA^mut^ LBC to induce 5‐LOX transcription and that PIK3CA^mut^ highly contributed to the development of immunosuppressive environment by recruiting MDSC infiltration.

### 3.6 Activation of the LTB4‐dependent 5‐LOX pathway contributes to MDSC recruitment and the immunosuppressive TIME in PIK3CA^mut^ LBC

**FIGURE 6:**
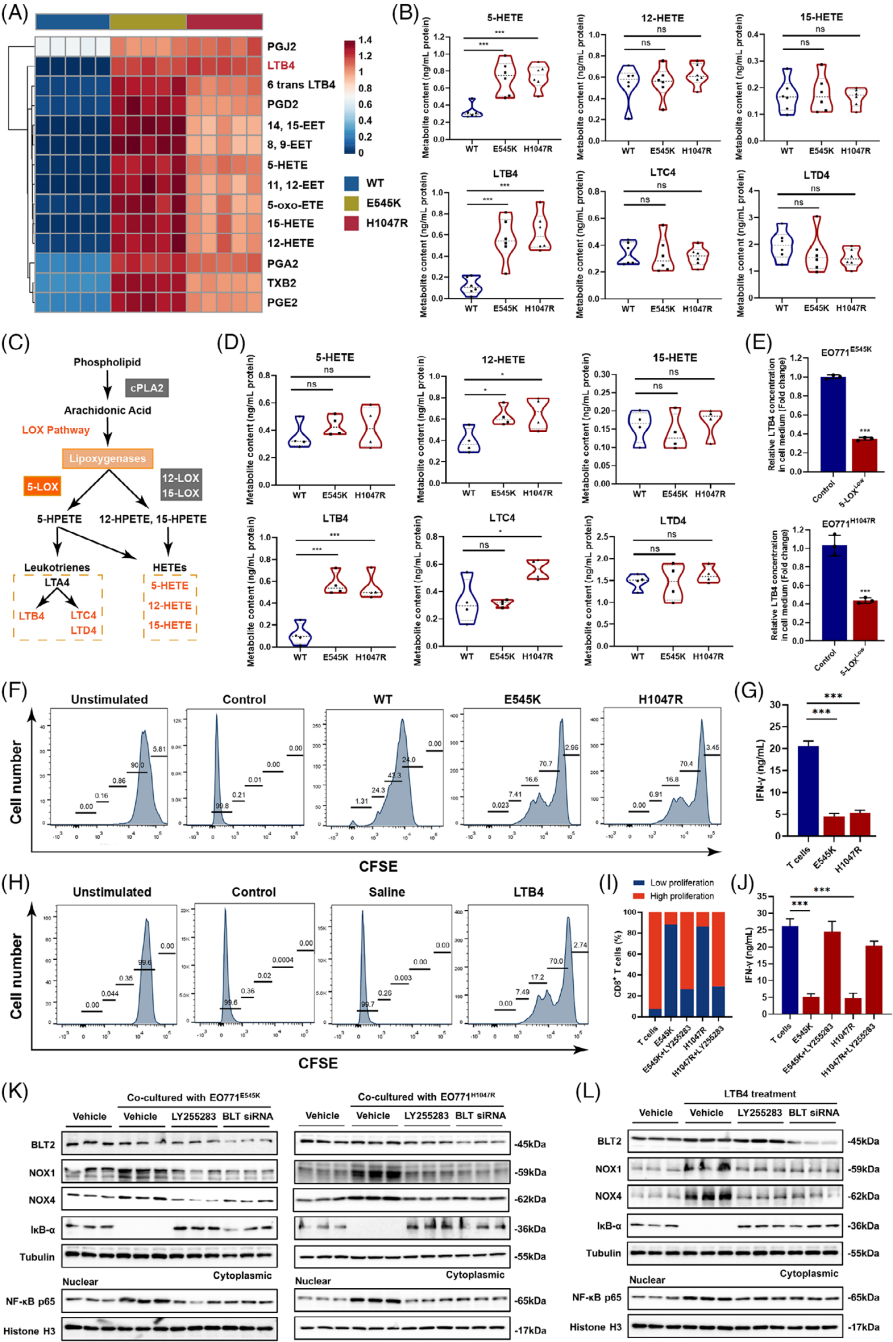
Activation of the LTB4‐dependent 5‐LOX pathway contributes to MDSC recruitment and immunosuppressive TIME in PIK3CA^mut^ LBC. (A) Heatmap of AA targeting metabolomics features. (B) ELISA results of AA pathway metabolites by the LOX pathway from cell medium, including 5‐HETE, 12‐HETE, 15‐HETE, LTB4, LTC4 and LTD4. (C) A model of the arachidonic acid metabolic pathway. (D) ELISA results of AA pathway metabolites by LOX pathway from tumour tissues. (E) ELISA results of LTB4 from EO771^E545K^ and EO771^H1047R^ cell medium treated with U73122. (F) The CFSE results of T‐cell proliferation in vitro. (G) The ELISA results of IFN‐γ from T cells. (H) The CFSE results of T‐cell proliferation in vitro treated with LTB4. (I) The CFSE results of T‐cell proliferation in vitro. (J) The ELISA results of IFN‐γ from T cells. (K) The protein expression of BLT2, NOX1, NOX4, IκB‐α and NF‐κB p65 was detected in MDSCs cultured with EO771 cells. (L) The protein expression of BLT2, NOX1, NOX4, IκB‐α and NF‐κB p65 was detected in MDSCs treated with LTB4. Data represent mean ± SD. *p < .05, **p < .01, ***p < .001.

As AA metabolites play integral roles in tumour immunity, especially in the heterotypic interactions between tumour and immune cells in the TIME,^31^ we audited the metabolome of PIK3CA^mut^ cells and found that the levels of 15 common AA pathway‐related metabolites in cell lysates, including LTB4, PGA2, PGE2, PGD2, 5‐HETE, 12‐HETE and 15‐HETE, increased dramatically, among which LTB4 was one of the most significantly upregulated metabolites (Figure 6A). Increased levels of 5‐HETE and LTB4 were detected in the supernatants of PIK3CA^mut^ cells compared to those of PIK3CA^wt^ cells (Figure 6B). As 5‐HETE and LTB4 are both metabolites of 5‐LOX (Figure 6C), we measured the levels of AA metabolites in PIK3CA^mut^ EO771 xenograft tissues and validated that the levels of LTB4 increased compared to PIK3CA^wt^ tissues (Figure 6D). Furthermore, when we applied U73122 to inhibit 5‐LOX enzyme activity in PIK3CA^mut^ cells, the level of LTB4 was highly repressed accordingly (Figure 6E). Then, we applied 740‐YP to stimulate PI3K enzyme activity in MCF‐10A and EO771 cells, and the level of LTB4 increased dramatically (Figure S4A). In contrast, when we applied alpelisib and CH5132799 to inhibit PI3K enzyme activity in MCF‐7 or T‐47D cells, the level of LTB4 was highly repressed accordingly (Figure S4B). To verify that the regulatory effect of PIK3CA^mut^ on AA metabolic reprogramming is mediated by 5‐LOX, we performed ELISA. We applied siRNA to inhibit 5‐LOX expression in PIK3CA^mut^ cells (EO771^E545K^ and EO771^H1047R^), and the level of AA was significantly repressed (Figure S4C).

As LTB4 is the ligand of BLT2, which acts as a potent chemotaxis to recruit dendritic cells in inflammation, we used the BLT2 antagonist LY255283 to investigate whether blocking the interaction between LTB4 and BLT2 had any effects on PIK3CA^mut^‐induced MDSC migration and function. First, we detected that MDSCs cocultured with PIK3CA^mut^ LBC cells showed stronger immunosuppression of T‐cell proliferation (Figure 6F) and IFN‐γ secretion (Figure 6G) than those cocultured with PIK3CA^wt^ cells. Similarly, we found that MDSCs pretreated with LTB4 effectively reduced T‐cell proliferation (Figure 6H). Accordingly, MDSC‐induced immunosuppression of T‐cell proliferation (Figure 6I) and IFN‐γ secretion (Figure 6J) was attenuated by an antagonist of BLT2, LY255283, in vitro. Furthermore, the expression of NOX1/4 and nuclear P65, the key downstream proteins of BLT2, was notably increased in MDSCs cocultured with PIK3CA^mut^ cells (Figure 6K). Such effects were abrogated by BLT2 knockdown or LTB4 antagonist (Figure 6K). Similar results were detected in LTB4‐pretreated MDSCs (Figure 6L). Furthermore, to verify the effect of LTB4 secreted from cancer cells on the migration and immunosuppressive role of MDSCs, we indirectly performed coculture assay. The conditioned media harvested from PIK3CA^mut^ cells treated with or without U73122 were used for the transwell chemotaxis assay. MDSCs cocultured with media collected from PIK3CA^mut^ cells treated with U73122 displayed less chemotactic activity in vitro (Figure S4D). We performed RNA‐seq to compare the most differentially expressed signalling pathways between LTB4‐pretreated MDSCs and controls, and found that the NF‐κB signalling pathway was one of the most significantly activated signalling pathways (Figure S4E), which was further validated by converse changes in IκBα protein in MDSCs either cocultured with PIK3CA^mut^ cells (Figure 6K) or pretreated with LTB4 (Figure 6L). Previous reports revealed that the NF‐κB signalling pathway plays a crucial role in the amplification and maintenance of the immunosuppression function of CD33^+^ MDSCs in breast cancer.^32^ Therefore, our findings indicated that activation of the LTB4‐dependent 5‐LOX pathway contributes to MDSC recruitment and immunosuppressive function in PIK3CA^mut^ LBC.

### 3.7 Targeted therapy against the PI3K/5‐LOX/LTB4 axis synergized with immune checkpoint blockade (ICB) therapy to enhance antitumour efficacy in vivo

**FIGURE 7:**
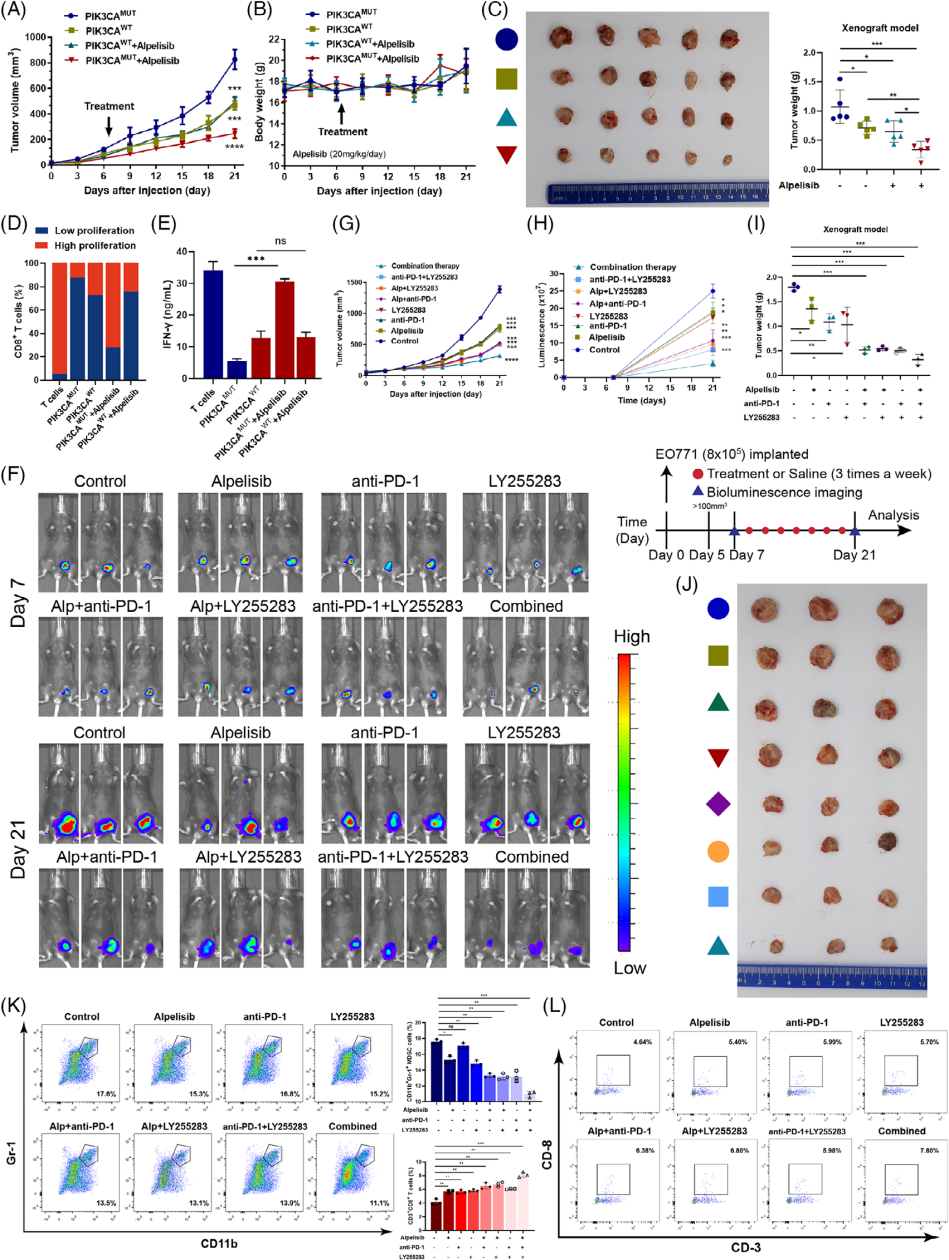
Targeted therapy against the PI3K/5‐LOX/LTB4 axis synergized with immune checkpoint blockade therapy to enhance antitumour efficacy in vivo. Tumour formation in female C57 mice (*n* = 5) injected subcutaneously with PIK3CA^wt^ or PIK3CA^mut^ EO771 cells as indicated. The tumour volume (A) and bodyweight (B) of the mice at various time points upon injection. (C) The mice with xenografts were administered with saline or alpelisib (20 mg/kg/day) until sacrifice. Then, representative photos of the indicated treated xenografts are presented. The size and weight of tumours were significantly reduced in the PIK3CA^mut^ treatment group. (D) MDSCs were sorted from the tumours of these mice and cocultured with normal T cells for 48 h. The harvested cells were analyzed for T‐cell proliferation and IFN‐γ secretion (E). Tumour formation in female C57 mice (*n* = 3) injected subcutaneously with PIK3CA^mut^ EO771 cells as indicated. (F) Representative tumour bioluminescence images of mice at 7 and 21 days after tumour implantation. (G) Tumour volume. (H) Tumour growth curves for mice by quantification of bioluminescent signal intensities. (I) Mice with xenografts were administered with alpelisib, LY255283 (10 mg/kg/day) or anti‐PD‐1 mAbs (10 mg/kg/day), alone or in combination until sacrifice. The size and weight of tumours were significantly reduced in the combined treatment group. (J) Representative photos of the indicated treated xenografts are presented. (K) Representative flow cytometry data showing the proportion of MDSCs and CD8^+^ T cells (L) isolated from the tumours. Data represent mean ± SD. *p < .05, **p < .01, ***p < .001.

The PIK3CA^wt^ or PIK3CA^mut^ EO771 cells were engrafted subcutaneously in female C57 mice until xenografts were established, followed by administration of either saline or alpelisib (20 mg/kg/day) afterwards. After 21 days, the average volume of PIK3CA^mut^ tumours in alpelisib‐treated group was much smaller than that of the other groups, while the PIK3CA^wt^ tumours were insensitive to alpelisib (Figure 7A). The bodyweight curves indicated that alpelisib did not impact physiological status during the treatment (Figure 7B). Similarly, the average weight of PIK3CA^mut^ tumours in the alpelisib‐treated group was much lower than that of the other groups (Figure 7C). Western blot analysis verified the decreased expression of p‐Akt (both S473 and T308), p‐STAT3 (both Y705 and S727) and 5‐LOX in alpelisib‐treated groups in vivo, which was consistent with the in vitro results (Figure S5A). The ELISA results revealed that the level of LTB4 in the alpelisib‐treated PIK3CA^mut^ group was much lower than that in the control group, while there was no difference between the alpelisib and control groups in PIK3CA^wt^ tumours (Figure S5B).

To determine whether infiltrated MDSCs in situ had an impact on LBC tumour growth, EO771 cells were engrafted in either C57 mice or C57^SOCS3KO^ mice, the latter of which were characterized by abundant tumour‐infiltrating MDSCs in previous research.^29^ As shown in Figure S5C, the growth of EO771 cells was significantly enhanced, indicating that MDSC recruitment (Figure S5D) potentially stimulated LBC carcinogenesis in vivo. Therefore, we compared the amounts of MDSCs in xenografts among different groups and found that MDSCs infiltrated in situ of alpelisib‐treated PIK3CA^mut^ group were reduced significantly compared to those in untreated PIK3CA^mut^ tissues (Figure S5E). The gating strategies for MDSCs and CD8^+^ T cells are shown in Figure S5F,G. Furthermore, MDSCs isolated from PIK3CA^mut^ tumours in the alpelisib‐treated group induced less inhibition of T‐cell proliferation (Figure 7D) and IFN‐γ secretion (Figure 7E) than those in the other groups, which indicated that the suppressive TIME in PIK3CA^mut^ LBC xenografts was attenuated by the PI3K inhibitor alpelisib.

To investigate whether blocking the PI3K/5‐LOX/LTB4 axis can inhibit the growth of PIK3CA^mut^ LBC and synergize with ICB therapy in vivo, we applied alpelisib, LY255283 (10 mg/kg/day) and an anti‐PD‐1 mAb (10 mg/kg/day) alone or in combination after PIK3CA^mut^ xenografts were established (Figure 7F). Compared with the controls, alpelisib, LY255283 and anti‐PD‐1 mAb alone or combined treatment significantly inhibited the growth (Figure 7G,H) and the weight of PIK3CA^mut^ xenografts (Figure 7I,J), especially the combination of alpelisib, LY255283 and anti‐PD‐1 mAb, which displayed the most significant suppression of tumour growth, suggesting that blocking the PI3K/5‐LOX/LTB4 axis by either PIK3CA inhibitor or LTB4 inhibitor considerably improved the efficacy of ICB therapy in PIK3CA^mut^ tumours. Pathological examination of HE staining revealed that the tumours had no lung metastases or liver metastases (Figure S6A). Furthermore, the recruitment of MDSCs decreased while the infiltration of CD8^+^ T cells increased in alpelisib, LY255283 or anti‐PD‐1 mAb group, especially in alpelisib, LY255283 and anti‐PD‐1 mAb combined group in which infiltrated MDSCs were the lowest (Figure 7K) and CD8^+^ T cells were the highest (Figure 7L). Immunohistochemical results showed that the expression of Granzyme, a marker involved in the activity of infiltrated T cells, was highest in the combination treatment group (Figure S6B). Flow cytometry assays revealed that the recruitment of M‐MDSCs (Figure S6C), PMN‐MDSCs (Figure S6D) and eMDSCs (Figure S6E) significantly decreased in the combined treatment groups, especially M‐MDSCs and eMDSCs. Thus, it seems that M‐MDSCs and eMDSCs are mainly involved in the interactions between LBC and TIME. Furthermore, MDSCs isolated from the combined group induced less inhibition of T‐cell proliferation (Figure S6F). Western blot analysis verified a decrease in the expression of p‐Akt (both S473 and T308), p‐STAT3 (both Y705 and S727) and 5‐LOX in the combined group in vivo (Figure S6G). ELISA confirmed that the level of LTB4 decreased significantly in alpelisib, alpelisib plus LY255283, alpelisib plus anti‐PD‐1 mAb and combined groups (Figure S6H). Additionally, MDSCs might express PD‐L1 to suppress T‐cell response by the PD‐1/PD‐L1 interaction.^33^ To validate whether the binding of LTB4 to BLT2 increases PD‐L1 expression on MDSCs, we compared the expression of PD‐L1 protein between PIK3CA^mut^ tumours treated with or without LY255283 by IHC. The results revealed that PD‐L1 is mainly expressed on tumour cells and that LY255283 did not affect the expression level of PD‐L1 on tumour cells (Figure S6I). However, less PD‐L1 was expressed on MDSCs in the LY255283‐treated group (Figure S6J). Thus, anti‐PD‐1 therapy restores T cells from an exhausted state and enhances their tumour‐killing activity in PIK3CA^mut^ LBC.

Furthermore, we confirmed if the drugs alpelisib and LY255283 mainly target on tumours. We knocked out BLT2 expression in PIK3CA^mut^ cells. BLT2^NC^ or BLT2^Knockout^ EO771 cells (PIK3CA^mut^) were engrafted subcutaneously in female C57 mice. After 21 days, the average weight of tumours in the BLT2^Knockout^ groups was not significantly different from that in the control groups (Figure S7A). Additionally, we applied gemtuzumab, an antibody drug targeting CD33^+^, to deplete MDSCs in TIME. The results revealed that the average weight and volume of tumours in alpelisib plus gemtuzumab‐treated group was much smaller than that of alpelisib‐treated group (Figure S7B,C). The results confirmed that MDSCs contributed to a greater effect of PIK3CA^mut^‐induced AA metabolism pathway activation in TIME. These findings demonstrated that the growth of PIK3CA^mut^ xenografts could be potently inhibited by targeted therapy against the PI3K/5‐LOX/LTB4 axis, which synergized with ICB therapy to enhance antitumour efficacy in vivo.

## 4 DISCUSSION

**FIGURE 8:**
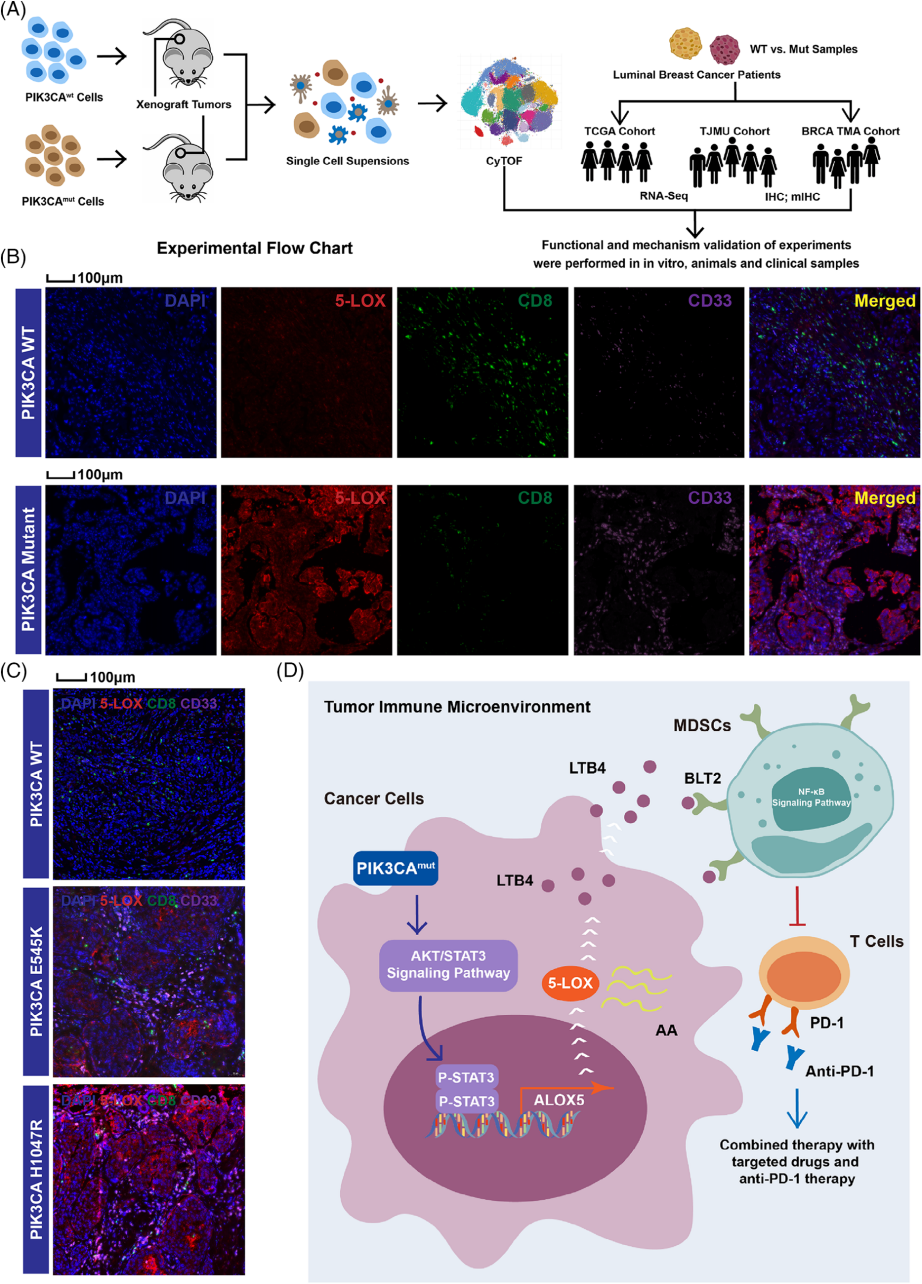
Oncogenic PIK3CA promotes a high MDSC infiltration immunosuppressive profile by activating 5‐LOX in LBC tumour tissues. (A) Experimental flowchart. (B) mIHC staining for 5‐LOX, MDSCs, T cells and tumour cells in BRCA TMA cohort. (C) mIHC staining for 5‐LOX, MDSCs, T cells and tumour cells in tumour tissues of LBC xenograft‐bearing mice. (D) Graph of a proposed model.

As PIK3CA is known to be one of the major driver oncogenes, the discovery of activating PIK3CA mutations has been elucidated in multiple cancers, including breast cancer, cervical cancer and lung cancer.^34^ The two hotspot mutation positions (E545K and H1047R) have been reported to be highly frequent in LBC.^10^ Significant preclinical evidence demonstrates a correlation between the vigorous stimulation of this pathway and the development of resistance to conventional treatments; however, fewer reports have focused on PIK3CA^mut^‐mediated TIME remodelling. Studies have shown that key molecular alterations in tumour cells can drive the formation of a specific TIME.^7^ Deletion of PTEN and mutations in PIK3CA can reduce the level of immune cell infiltration in TIME by downregulating IFN‐α/β.^6^, ^35^ Amplification of MYC can reduce T‐cell activity and promote polarization of M2 macrophages through the upregulation of PD‐L1 and CD47.^17^ Therefore, in this study, we investigated primary tumour tissues, tumour cell lines and xenograft‐bearing mouse models to elucidate the mechanisms involved in the development of the PIK3CA^mut^‐related suppressive TIME (Figure 8A).

Our study showed that a suppressive TIME in PIK3CA^mut^ LBC was featured with higher MDSC infiltration, which was recruited by AA metabolism reprogramming. MDSCs are important players in the suppressive TIME that may contribute to patient resistance to ICB therapy.^36^, ^37^ Previous studies have shown that these cells are abundant in TIME, where multiple inflammatory cytokines and chemokines might lead to the recruitment of MDSCs to tumour tissues.^38^ However, the mechanisms that activate MDSC function in TIME remain unclear. The JAK/STAT3 pathway and NF‐κB pathways are crucial for the immunosuppressive function of MDSCs.^32^, ^39^ Recent works have highlighted the importance of metabolic reprogramming in regulating MDSC function within TIME.^40^, ^41^, ^42^ It was shown previously that lipid metabolism is a crucial factor in modulating MDSC function.^40^, ^42^ In addition, a recent study found that PIK3CA^mut^ could stimulate lipid metabolism reprogramming and shape the immune microenvironment.^41^ Therefore, we compared both the transcriptome and metabolome between PIK3CA^mut^ and PIK3CA^wt^ LBCs to define whether metabolic reprogramming is involved in PIK3CA^mut^‐related MDSC recruitment in TIME.

We found that higher expression of 5‐LOX was correlated with more MDSCs and less T‐cell infiltration in human PIK3CA^mut^ LBC tissues than in PIK3CA^wt^ tissues (Figure 8B). Similarly, higher amounts of intra‐tumour MDSCs, lower amounts of intra‐tumour T cells and higher levels of 5‐LOX expression were observed in PIK3CA^mut^ LBC xenografts in vivo (Figure 8C). Consistent results were observed in LBC tissue samples from BRCA TMA cohort using the mIHC assay (Figure S7D). Indeed, in this study, we found that a total of 36 adhesion‐, apoptosis‐, metabolism‐ and immunology‐related pathways were upregulated in PIK3CA^mut^ tumours. Metabolic reprogramming has been reported as a key trigger of tumour evasion from T‐cell surveillance, and our previous studies demonstrated that MDSCs induced T‐cell immunosuppression in breast cancer.^21^, ^43^ Therefore, we focused on metabolism‐related pathways during the cross‐talk between PIK3CA^mut^ LBC and MDSCs. We used Venn diagrams to take the intersection of upregulated pathways related to PIK3CA^mut^ tumours in the TCGA cohort and TJMU cohort, as well as MDSC high infiltration‐related pathways, thus obtaining 33 targets. Among the enriched pathways, we found that the AA pathway was exclusively the only metabolism‐related pathway. In a previous study, it was reported that PIK3CA mutations appeared to be linked to AA metabolism in breast cancer, revealing a potential role of the AA pathway in the tumour microenvironment.^41^ Combined with in vitro and in vivo experiments, we demonstrated that PIK3CA^mut^ initiated AA metabolism reprogramming in LBC by activating the 5‐LOX/LTB4 axis in STAT3‐dependent manner, which further recruited MDSC infiltration and inhibited the antitumour efficacy of T cells (Figure 8D).

AAs play a central role in a variety of diseases, including cancers, diabetes, asthma and autoimmune diseases.^44^ In particular, AA metabolites play an integral role in tumours, especially in the heterotypic interactions between tumour cells and immune cells in TIME.^31^ Enhanced PGE2 production from the tumour hinders the complete activation of CD8^+^ T cells, thereby facilitating immune evasion.^45^, ^46^ Moreover, the application of cPLA2 inhibitors enhances the infiltration of activated and proliferating CD4^+^ and CD8^+^ T cells in TIME of aggressive breast cancer.^47^ Activation of 5‐LOX leads to a proinflammatory environment that induces tumour development.^48^ Considering that high expression of 5‐LOX resulted in LTB4 upregulation^49^ and persistent elevation of LTB4 has been reported in cancer,^30^ PIK3CA^mut^ activating the 5‐LOX/LTB4 axis in LBC cells not only aggravated the suppressive TIME but also hampered the efficacy of immunotherapy by facilitating the migration and function of MDSCs.

ICB therapy has developed rapidly, and has been proven to provide clinical benefits to multiple cancer patients, including in breast cancer. However, the efficacy of the treatment is still controversial, and not all LBC patients benefit equally from ICB therapy.^50^ The vast majority of LBC do not respond to therapy. Oncogenic PIK3CA mutations frequently occur in a higher proportion in LBC, especially in refractory advanced patients, which predicts unfavourable efficacy of immunotherapy in cancers.^51^ Although PIK3CA mutation could induce PD‐L1 expression on cancer cells, it has also been reported to induce a suppressive TIME, a critical barrier for the success of cancer immunotherapy.^52^, ^53^ Therefore, elucidating how PIK3CA mutation regulates the suppressive TIME in LBC will help to enhance the antitumour efficacy of immunotherapy.

Recent studies have shown that multiple immunocytes infiltrating in TIME affect the efficacy of immunotherapy,^54^ among which MDSCs contribute to immune suppression and limit the effectiveness of immunotherapies.^27^, ^55^ Therefore, reversing the suppressive TIME is a promising strategy for improving immunotherapy outcomes.^56^ Our study indicated that PIK3CA^mut^ can induce immune evasion of LBC by recruiting MDSCs through the 5‐LOX‐dependent AA pathway, and a PI3K inhibitor could synergize with ICB therapy by systematically recovering the immune competent TIME by excluding MDSCs and recruiting cytotoxic T cells. The results provide strategies that can convert ‘cold’ tumours into ‘hot’ tumours in LBC in vivo, which might provide a promising treatment option for refractory advanced LBC patients. However, 5‐LOX‐dependent AA metabolism might not play a critical role in the basal subtypes of breast cancer, and the molecular mechanism of the metabolic reprogramming process is still a challenge due to the complexity of basal‐like breast cancer.

This finding was consistent with a previous study indicating that modulation of AA levels in PIK3CA^mut^ tumours by cPLA2 inhibition and dietary fat restriction increased intra‐tumour infiltration of NK cells.^41^ Similarly, multiple clinical trials have recently combined MDSC‐depleting strategies with immune checkpoint inhibitors,^57^ and using combined ICB therapies for breast cancers has improved therapeutic efficacy in the clinic.^58^, ^59^ Therefore, we hope that this work may shed light on the promising future of combined ICB treatment for refractory advanced LBC patients.

## CONFLICT OF INTEREST STATEMENT

The authors declare they have no conflicts of interest.

## Supporting information

Supporting Information

## Data Availability

The datasets involved in the study were extracted from TCGA dataset (https://portal.gdc.cancer.gov/repository), cBioportal (https://www.cbioportal.org/) and GEO dataset (https://www.ncbi.nlm.nih.gov/geo). The datasets used and/or analyzed during the current study are available from the corresponding author upon reasonable request.

## References

[1] Ignatiadis M , Sotiriou C . Luminal breast cancer: from biology to treatment. Nat Rev Clin Oncol. 2013;10(9):494‐506. doi:10.1038/nrclinonc.2013.124 23881035

[2] Farrance I , Aldons J . Paracetamol interference with YSI glucose analyzer. Clin Chem. 1981;27(5):782‐783. 7226518

[3] Clarke R , Tyson JJ , Dixon JM . Endocrine resistance in breast cancer—an overview and update. Mol Cell Endocrinol. 2015;418:220‐234. doi:10.1016/j.mce.2015.09.035 26455641 PMC4684757

[4] Xing Y , Lin NU , Maurer MA , et al. Phase II trial of AKT inhibitor MK‐2206 in patients with advanced breast cancer who have tumors with PIK3CA or AKT mutations, and/or PTEN loss/PTEN mutation. Breast Cancer Res. 2019;21(1):78. doi:10.1186/s13058-019-1154-8 31277699 PMC6612080

[5] Miricescu D , Totan A , Stanescu S II , Badoiu SC , Stefani C , Greabu M . PI3K/AKT/mTOR signaling pathway in breast cancer: from molecular landscape to clinical aspects. Int J Mol Sci. 2020;22(1). doi:10.3390/ijms22010173 PMC7796017 33375317

[6] Sai J , Owens P , Novitskiy SV , et al. PI3K inhibition reduces mammary tumor growth and facilitates antitumor immunity and anti‐PD1 responses. Clin Cancer Res. 2017;23(13):3371‐3384. doi:10.1158/1078-0432.CCR-16-2142 28003307 PMC5479746

[7] Wellenstein MD , de Visser KE . Cancer‐cell‐intrinsic mechanisms shaping the tumor immune landscape. Immunity. 2018;48(3):399‐416. doi:10.1016/j.immuni.2018.03.004 29562192

[8] Yan C , Yang J , Saleh N , et al. Inhibition of the PI3K/mTOR pathway in breast cancer to enhance response to immune checkpoint inhibitors in breast cancer. Int J Mol Sci. 2021;22(10):5207. doi:10.3390/ijms22105207 34069042 PMC8156389

[9] Pan JW , Zabidi MMA , Ng PS , et al. The molecular landscape of Asian breast cancers reveals clinically relevant population‐specific differences. Nat Commun. 2020;11(1):6433. doi:10.1038/s41467-020-20173-5 33353943 PMC7755902

[10] Lang GT , Jiang YZ , Shi JX , et al. Characterization of the genomic landscape and actionable mutations in Chinese breast cancers by clinical sequencing. Nat Commun. 2020;11(1):5679. doi:10.1038/s41467-020-19342-3 33173047 PMC7656255

[11] Martinez‐Saez O , Chic N , Pascual T , et al. Frequency and spectrum of PIK3CA somatic mutations in breast cancer. Breast Cancer Res. 2020;22(1):45. doi:10.1186/s13058-020-01284-9 32404150 PMC7222307

[12] Andre F , Ciruelos E , Rubovszky G , et al. Alpelisib for PIK3CA‐mutated, hormone receptor‐positive advanced breast cancer. N Engl J Med. 2019;380(20):1929‐1940. doi:10.1056/NEJMoa1813904 31091374

[13] Vanhaesebroeck B , Burke JE , Madsen RR . Precision targeting of mutant PI3Kalpha in cancer by selective degradation. Cancer Discov. 2022;12(1):20‐22. doi:10.1158/2159-8290.CD-21-1411 35022207 PMC7612218

[14] Brufsky AM , Dickler MN . Estrogen receptor‐positive breast cancer: exploiting signaling pathways implicated in endocrine resistance. Oncologist. 2018;23(5):528‐539. doi:10.1634/theoncologist.2017-0423 29352052 PMC5947450

[15] Okkenhaug K , Graupera M , Vanhaesebroeck B . Targeting PI3K in cancer: impact on tumor cells, their protective stroma, angiogenesis, and immunotherapy. Cancer Discov. 2016;6(10):1090‐1105. doi:10.1158/2159-8290.CD-16-0716 27655435 PMC5293166

[16] Crowley MJP , Bhinder B , Markowitz GJ , et al. Tumor‐intrinsic IRE1alpha signaling controls protective immunity in lung cancer. Nat Commun. 2023;14(1):120. doi:10.1038/s41467-022-35584-9 36624093 PMC9829901

[17] Casey SC , Tong L , Li Y , et al. MYC regulates the antitumor immune response through CD47 and PD‐L1. Science. 2016;352(6282):227‐231. doi:10.1126/science.aac9935 26966191 PMC4940030

[18] Tesi RJ . MDSC; the most important cell you have never heard of. Trends Pharmacol Sci. 2019;40(1):4‐7. doi:10.1016/j.tips.2018.10.008 30527590

[19] Bronte V , Brandau S , Chen SH , et al. Recommendations for myeloid‐derived suppressor cell nomenclature and characterization standards. Nat Commun. 2016;7:12150. doi:10.1038/ncomms12150 27381735 PMC4935811

[20] Umansky V , Blattner C , Fleming V , et al. Myeloid‐derived suppressor cells and tumor escape from immune surveillance. Semin Immunopathol. 2017;39(3):295‐305. doi:10.1007/s00281-016-0597-6 27787613

[21] Jiang M , Zhang W , Zhang R , et al. Cancer exosome‐derived miR‐9 and miR‐181a promote the development of early‐stage MDSCs via interfering with SOCS3 and PIAS3 respectively in breast cancer. Oncogene. 2020;39(24):4681‐4694. doi:10.1038/s41388-020-1322-4 32398867

[22] Chen G , Li X , Ji C , et al. Early myeloid‐derived suppressor cells accelerate epithelial‐mesenchymal transition by downregulating ARID1A in luminal A breast cancer. Front Bioeng Biotechnol. 2022;10:973731. doi:10.3389/fbioe.2022.973731 36329699 PMC9623091

[23] Chen SMY , Li B , Nicklawsky AG , et al. Deletion of p53 and hyper‐activation of PIK3CA in keratin‐15(+) stem cells lead to the development of spontaneous squamous cell carcinoma. Int J Mol Sci. 2020;21(18):6585. doi:10.3390/ijms21186585 32916850 PMC7554792

[24] Zhang Y , Zhang Z . The history and advances in cancer immunotherapy: understanding the characteristics of tumor‐infiltrating immune cells and their therapeutic implications. Cell Mol Immunol. 2020;17(8):807‐821. doi:10.1038/s41423-020-0488-6 32612154 PMC7395159

[25] Nguyen PHD , Ma S , Phua CZJ , et al. Intratumoural immune heterogeneity as a hallmark of tumour evolution and progression in hepatocellular carcinoma. Nat Commun. 2021;12(1):227. doi:10.1038/Xs41467-020-20171-7 33431814 PMC7801667

[26] Xu L , Zou C , Zhang S , et al. Reshaping the systemic tumor immune environment (STIE) and tumor immune microenvironment (TIME) to enhance immunotherapy efficacy in solid tumors. J Hematol Oncol. 2022;15(1):87. doi:10.1186/s13045-022-01307-2 35799264 PMC9264569

[27] Liao W , Overman MJ , Boutin AT , et al. KRAS‐IRF2 axis drives immune suppression and immune therapy resistance in colorectal cancer. Cancer Cell. 2019;35(4):559‐572. doi:10.1016/j.ccell.2019.02.008 30905761 PMC6467776

[28] Mungenast F , Fernando A , Nica R , et al. Next‐generation digital histopathology of the tumor microenvironment. Genes (Basel). 2021;12(4):538. doi:10.3390/genes12040538 33917241 PMC8068063

[29] Zhang W , Li X , Jiang M , et al. SOCS3 deficiency‐dependent autophagy repression promotes the survival of early‐stage myeloid‐derived suppressor cells in breast cancer by activating the Wnt/mTOR pathway. J Leukoc Biol. 2023;113(5):445‐460. doi:10.1093/jleuko/qiad020 36808484

[30] Lin Y , Cai Q , Chen Y , et al. CAFs shape myeloid‐derived suppressor cells to promote stemness of intrahepatic cholangiocarcinoma through 5‐lipoxygenase. Hepatology. 2022;75(1):28‐42. doi:10.1002/hep.32099 34387870

[31] Sun Z , Zhang R , Zhang X , et al. LINE‐1 promotes tumorigenicity and exacerbates tumor progression via stimulating metabolism reprogramming in non‐small cell lung cancer. Mol Cancer. 2022;21(1):147. doi:10.1186/s12943-022-01618-5 35842613 PMC9288060

[32] Yu J , Wang Y , Yan F , et al. Noncanonical NF‐kappaB activation mediates STAT3‐stimulated IDO upregulation in myeloid‐derived suppressor cells in breast cancer. J Immunol. 2014;193(5):2574‐2586. doi:10.4049/jimmunol.1400833 25063873 PMC4719564

[33] Hsieh CH , Jian CZ , Lin LI , et al. Potential role of CXCL13/CXCR5 signaling in immune checkpoint inhibitor treatment in cancer. Cancers (Basel). 2022;14(2):294. doi:10.3390/cancers14020294 35053457 PMC8774093

[34] Zhang M , Jang H , Nussinov R . PI3K inhibitors: review and new strategies. Chem Sci. 2020;11(23):5855‐5865. doi:10.1039/d0sc01676d 32953006 PMC7472334

[35] Peng W , Chen JQ , Liu C , et al. Loss of PTEN promotes resistance to T cell‐mediated immunotherapy. Cancer Discov. 2016;6(2):202‐216. doi:10.1158/2159-8290.CD-15-0283 26645196 PMC4744499

[36] Davis RJ , Moore EC , Clavijo PE , et al. Anti‐PD‐L1 efficacy can be enhanced by inhibition of myeloid‐derived suppressor cells with a selective inhibitor of PI3Kdelta/gamma. Cancer Res. 2017;77(10):2607‐2619. doi:10.1158/0008-5472.CAN-16-2534 28364000 PMC5466078

[37] Weber R , Fleming V , Hu X , et al. Myeloid‐derived suppressor cells hinder the anti‐cancer activity of immune checkpoint inhibitors. Front Immunol. 2018;9:1310. doi:10.3389/fimmu.2018.01310 29942309 PMC6004385

[38] Latifi A , Ghanizadeh‐Vesali S , Hosseini S , Mohsenzadegan M . Clinical significance of peripheral blood CD11b^+^/CD33^+^/HLA^−^DR^−^ myeloid cells in infants and children with infectious diseases and increased CRP. Med J Islam Repub Iran. 2020;34:92. 10.34171/mjiri.34.92 33315975 PMC7722956

[39] Trikha P , Plews RL , Stiff A , et al. Targeting myeloid‐derived suppressor cells using a novel adenosine monophosphate‐activated protein kinase (AMPK) activator. Oncoimmunology. 2016;5(9):e1214787. doi:10.1080/2162402X.2016.1214787 27757311 PMC5048767

[40] Mohammadpour H , MacDonald CR , McCarthy PL , Abrams SI , Repasky EA . Beta2‐adrenergic receptor signaling regulates metabolic pathways critical to myeloid‐derived suppressor cell function within the TME. Cell Rep. 2021;37(4):109883. doi:10.1016/j.celrep.2021.109883 34706232 PMC8601406

[41] Koundouros N , Karali E , Tripp A , et al. Metabolic fingerprinting links oncogenic PIK3CA with enhanced arachidonic acid‐derived eicosanoids. Cell. 2020;181(7):1596‐1611.e27. doi:10.1016/j.cell.2020.05.053 32559461 PMC7339148

[42] Adeshakin AO , Liu W , Adeshakin FO , et al. Regulation of ROS in myeloid‐derived suppressor cells through targeting fatty acid transport protein 2 enhanced anti‐PD‐L1 tumor immunotherapy. Cell Immunol. 2021;362:104286. doi:10.1016/j.cellimm.2021.104286 33524739

[43] Zhang W , Jiang M , Chen J , et al. SOCS3 suppression promoted the recruitment of CD11b(+)Gr‐1(‐)F4/80(‐)MHCII(‐) early‐stage myeloid‐derived suppressor cells and accelerated interleukin‐6‐related tumor invasion via affecting myeloid differentiation in breast cancer. Front Immunol. 2018;9:1699. doi:10.3389/fimmu.2018.01699 30083161 PMC6064721

[44] Sonnweber T , Pizzini A , Nairz M , Weiss G , Tancevski I . Arachidonic acid metabolites in cardiovascular and metabolic diseases. Int J Mol Sci. 2018;19(11):3285. doi:10.3390/ijms19113285 30360467 PMC6274989

[45] Basingab FS , Ahmadi M , Morgan DJ . IFNgamma‐dependent interactions between ICAM‐1 and LFA‐1 counteract prostaglandin E2‐mediated inhibition of antitumor CTL responses. Cancer Immunol Res. 2016;4(5):400‐411. doi:10.1158/2326-6066.CIR-15-0146 26928462

[46] Go JH , Wei JD , Park JI , Ahn KS , Kim JH . Wogonin suppresses the LPS‑enhanced invasiveness of MDA‑MB‑231 breast cancer cells by inhibiting the 5‑LO/BLT2 cascade. Int J Mol Med. 2018;42(4):1899‐1908. doi:10.3892/ijmm.2018.3776 30015917 PMC6108877

[47] Mishra S , Charan M , Shukla RK , et al. cPLA2 blockade attenuates S100A7‐mediated breast tumorigenicity by inhibiting the immunosuppressive tumor microenvironment. J Exp Clin Cancer Res. 2022;41(1):54. doi:10.1186/s13046-021-02221-0 35135586 PMC8822829

[48] Weigert A , Strack E , Snodgrass RG , Brune B . mPGES‐1 and ALOX5/‐15 in tumor‐associated macrophages. Cancer Metastasis Rev. 2018;37(2‐3):317‐334. doi:10.1007/s10555-018-9731-3 29808459

[49] Brock TG , McNish RW , Bailie MB , Peters‐Golden M . Rapid import of cytosolic 5‐lipoxygenase into the nucleus of neutrophils after in vivo recruitment and in vitro adherence. J Biol Chem. 1997;272(13):8276‐8280. doi:10.1074/jbc.272.13.8276 9079648

[50] Luen S , Virassamy B , Savas P , Salgado R , Loi S . The genomic landscape of breast cancer and its interaction with host immunity. Breast. 2016;29:241‐250. doi:10.1016/j.breast.2016.07.015 27481651

[51] Mosele F , Stefanovska B , Lusque A , et al. Outcome and molecular landscape of patients with PIK3CA‐mutated metastatic breast cancer. Ann Oncol. 2020;31(3):377‐386. doi:10.1016/j.annonc.2019.11.006 32067679

[52] Schmid P , Adams S , Rugo HS , et al. Atezolizumab and nab‐paclitaxel in advanced triple‐negative breast cancer. N Engl J Med. 2018;379(22):2108‐2121. doi:10.1056/NEJMoa1809615 30345906

[53] Vesely MD , Zhang T , Chen L . Resistance mechanisms to anti‐PD cancer immunotherapy. Annu Rev Immunol. 2022;40:45‐74. doi:10.1146/annurev-immunol-070621-030155 35471840

[54] Cui J , Zheng L , Zhang Y , Xue M . Bioinformatics analysis of DNMT1 expression and its role in head and neck squamous cell carcinoma prognosis. Sci Rep. 2021;11(1):2267. doi:10.1038/s41598-021-81971-5 33500531 PMC7838186

[55] Zhang D , Baldwin P , Leal AS , Carapellucci S , Sridhar S , Liby KT . A nano‐liposome formulation of the PARP inhibitor Talazoparib enhances treatment efficacy and modulates immune cell populations in mammary tumors of BRCA‐deficient mice. Theranostics. 2019;9(21):6224‐6238. doi:10.7150/thno.36281 31534547 PMC6735511

[56] Miao L , Qi J , Zhao Q , et al. Targeting the STING pathway in tumor‐associated macrophages regulates innate immune sensing of gastric cancer cells. Theranostics. 2020;10(2):498‐515. doi:10.7150/thno.37745 31903134 PMC6929973

[57] Doi M , Tanaka H , Ohoto T , et al. Reactivation of anticancer immunity by resetting interorgan crosstalk in immune‐suppressive cells with a nanoparticulated anti‐inflammatory drug. Small. 2023:e2205131. doi:10.1002/smll.202205131 36703512

[58] Goel S , DeCristo MJ , Watt AC , et al. CDK4/6 inhibition triggers anti‐tumour immunity. Nature. 2017;548(7668):471‐475. doi:10.1038/nature23465 28813415 PMC5570667

[59] Schaer DA , Beckmann RP , Dempsey JA , et al. The CDK4/6 inhibitor abemaciclib induces a T cell inflamed tumor microenvironment and enhances the efficacy of PD‐L1 checkpoint blockade. Cell Rep. 2018;22(11):2978‐2994. doi:10.1016/j.celrep.2018.02.053 29539425

