# Supplementary material for: Oncogenic PIK3CA recruits myeloid‐derived suppressor cells to shape the immunosuppressive tumour microenvironment in luminal breast cancer through the 5‐lipoxygenase‐dependent arachidonic acid pathway
Source: Clin Transl Med. 2023 Nov 15;13(11):e1483. doi: 10.1002/ctm2.1483 (PMC10646754; doi:10.1002/ctm2.1483)
Supplement: Supplementary file 1 — Supporting Information [file CTM2-13-e1483-s001.docx]

**Supplementary Files**

**Supplementary Figures**

**
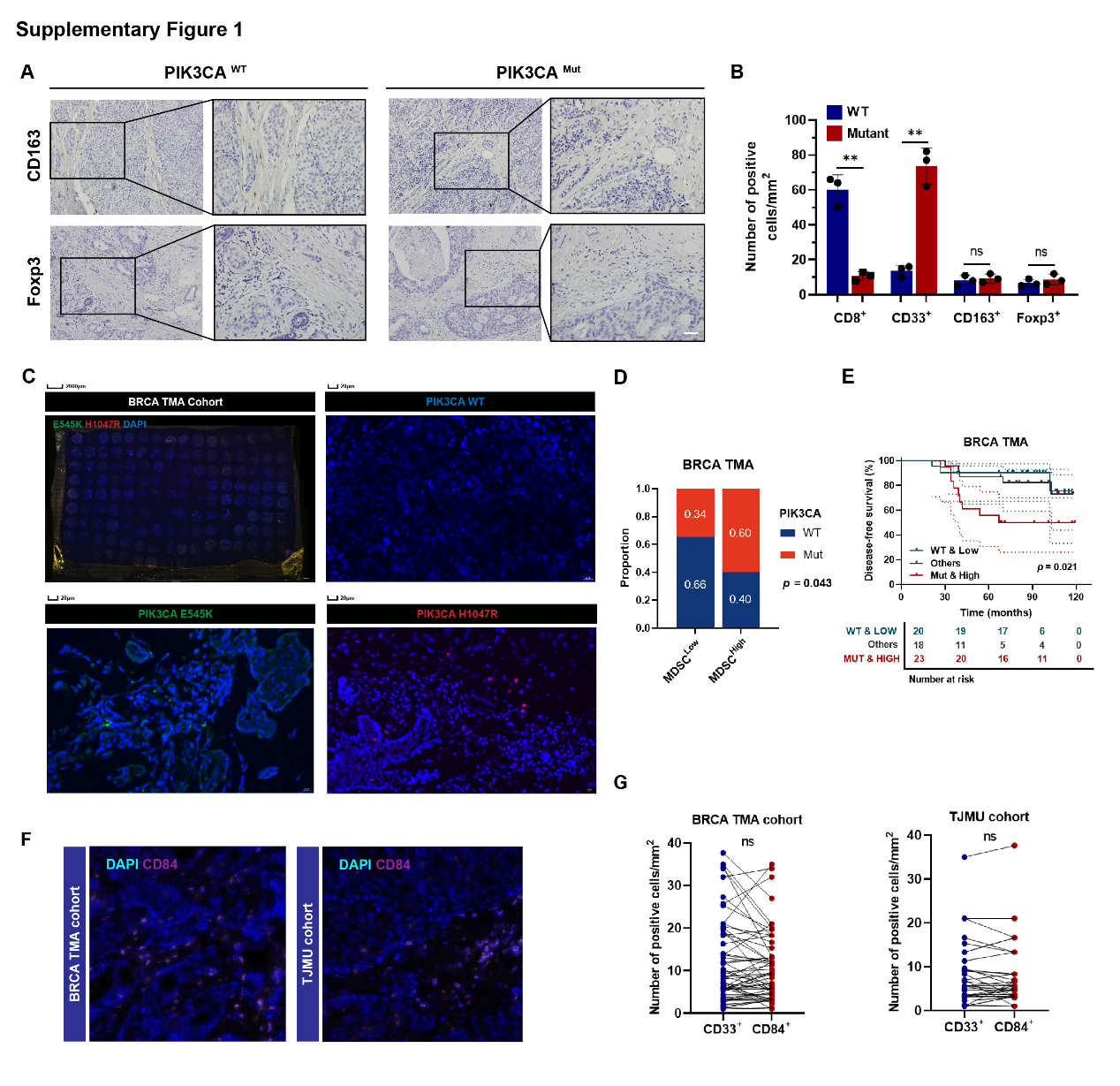
**

**Figure S1.** The tumor microenvironment in PIK3CA^mut^ tumors. (A) IHC analysis for CD163^+^ (M2 macrophages) and Foxp3 (Tregs) markers in tumor tissues and statistical graphs (B). Scale bar, 100 μm. (C) RCA assay revealed the mutant of PIK3CA gene in BRCA TMA cohort. (D) The results of correlation and the survival analysis (E) in the BRCA TMA cohort. (F) mIHC staining for CD84 positive cells in TJMU cohort and BRCA TMA cohort. (G) The *t*-test analysis showed no difference between the amount of CD33^+^ cells and CD84^+^ cells in LBC tissues. Data represent mean ± SD, **P* < 0.05, ***P* < 0.01, ****P* < 0.001.


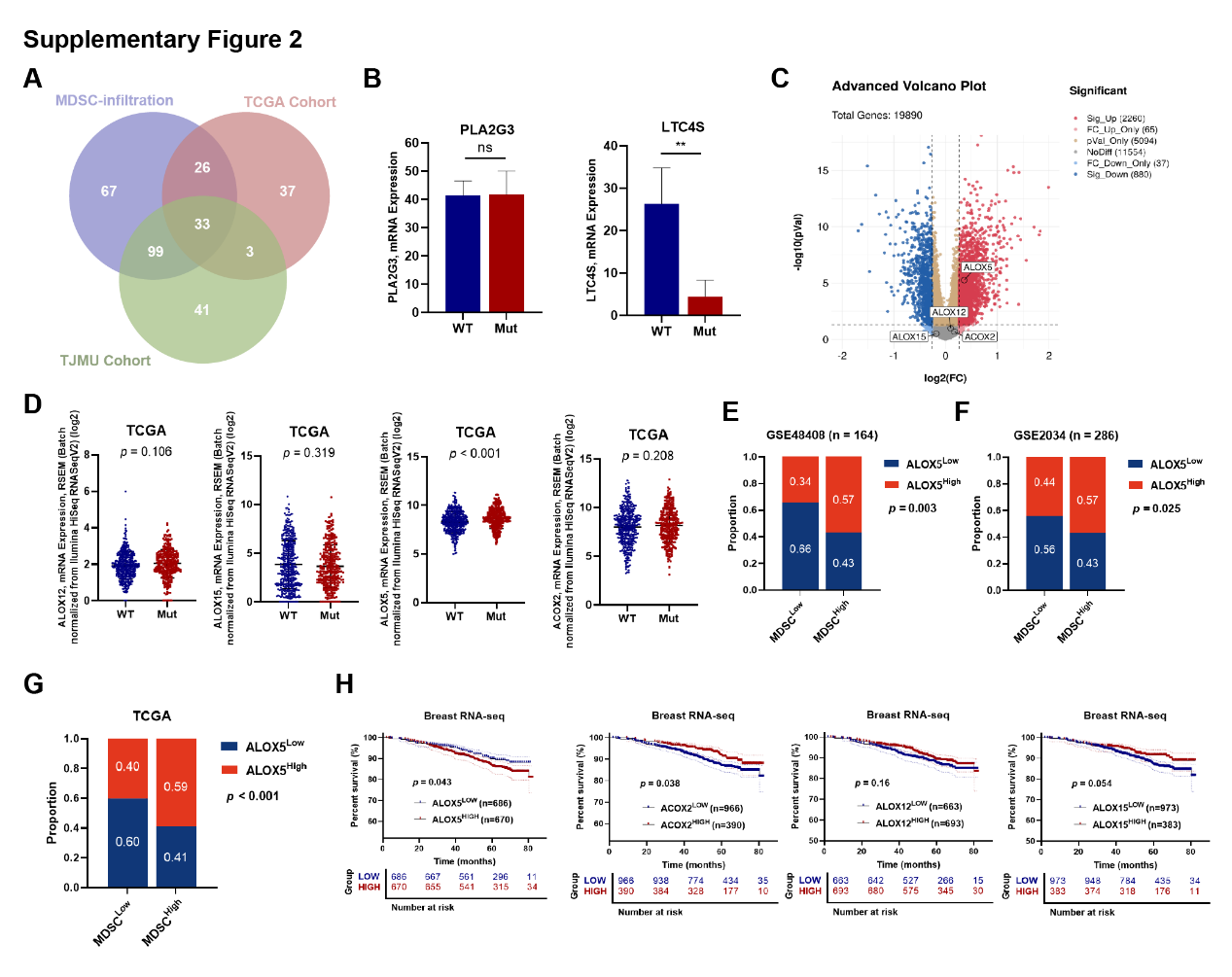


**Figure S2.** Analysis of TCGA database. (A) Venn diagram showed that 33 pathways were shared by all three groups. (B) The mRNA expression of PLA2G3 and LTC4S in GSE216871 dataset. (C) Volcano plot for differential gene expression. (D) The mRNA expression of ALOX5, ACOX2, ALOX12 and ALOX15 in TCGA cohort. (E) The overexpression of 5-LOX was positive correlated with the MDSCs infiltration in GSE48408, GSE2034 (F), and TCGA datasets (G). (H) Survival curve of LBC patients grouped as ALOX5, ACOX2, ALOX12 or ALOX15 high and low by the total expression of survival. Dataset: Kaplan-Meier plotter. Data represent mean ± SD, **P* < 0.05, ***P* < 0.01, ****P* < 0.001.


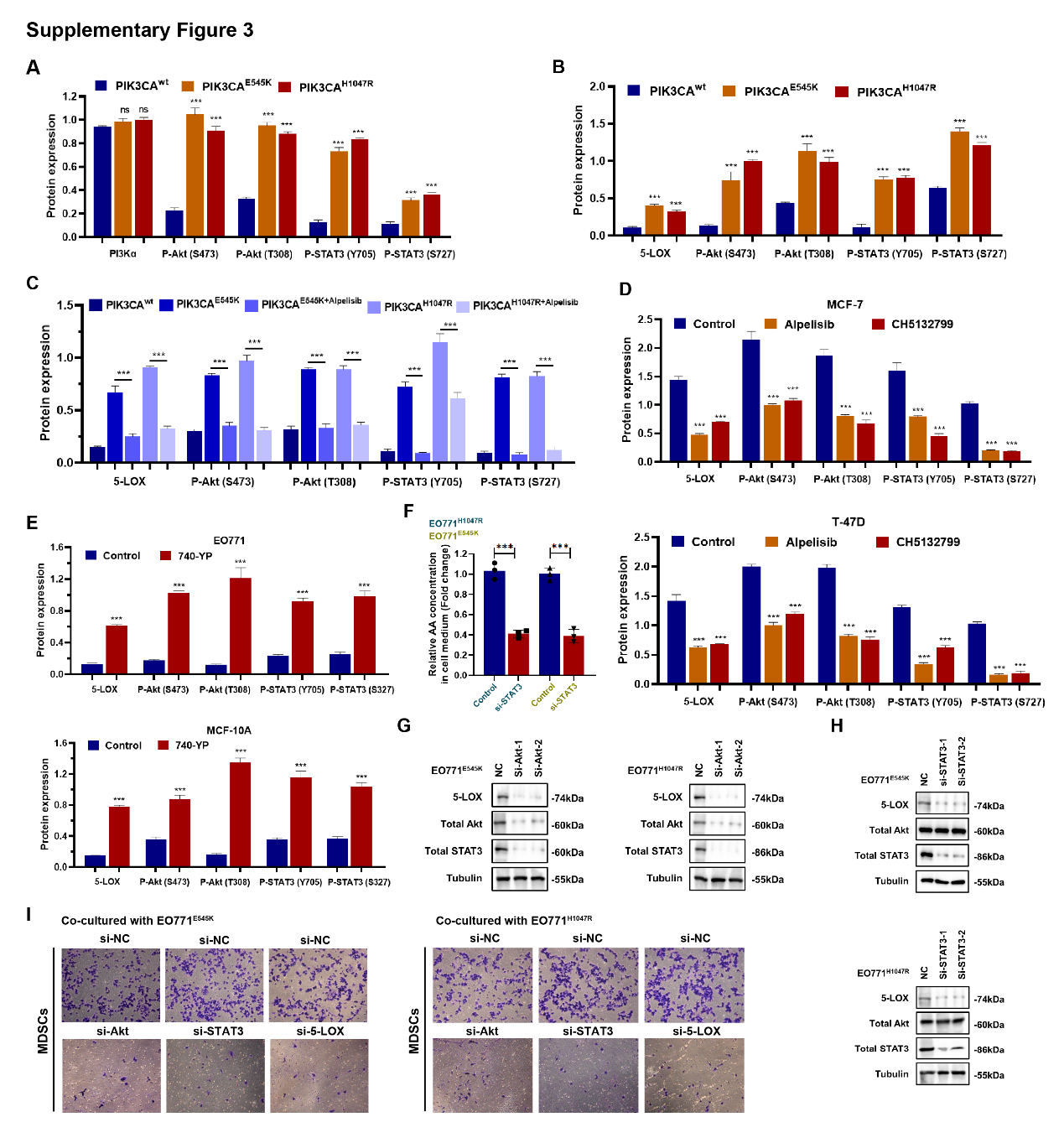


**Figure S3.** The oncogene PIK3CA^mut^ activated Akt/STAT3/5-LOX axis and recruited MDSCs infiltration. (A) The statistical analysis for the gray values of the protein bands in Figure 4D. (B) The statistical analysis for the gray values of the protein bands in Figure 4E. (C) The statistical analysis for the gray values of the protein bands in Figure 4F. (D) The statistical analysis for the gray values of the protein bands in Figure 4G. (E) The statistical analysis for the gray values of the protein bands in Figure 4H. (F) The ELISA results of AA from EO771^E545K^ and EO771^H1047R^ cell supernatants. (G) Western blot analysis of 5-LOX, Total Akt, and Total STAT3 for EO771^E545K^ and EO771^H1047R^ cells treated with si-Akt and si-STAT3 (H). (I) The transwell chemotaxis assay *in vitro*. Data represent mean ± SD, **P* < 0.05, ***P* < 0.01, ****P* < 0.001.


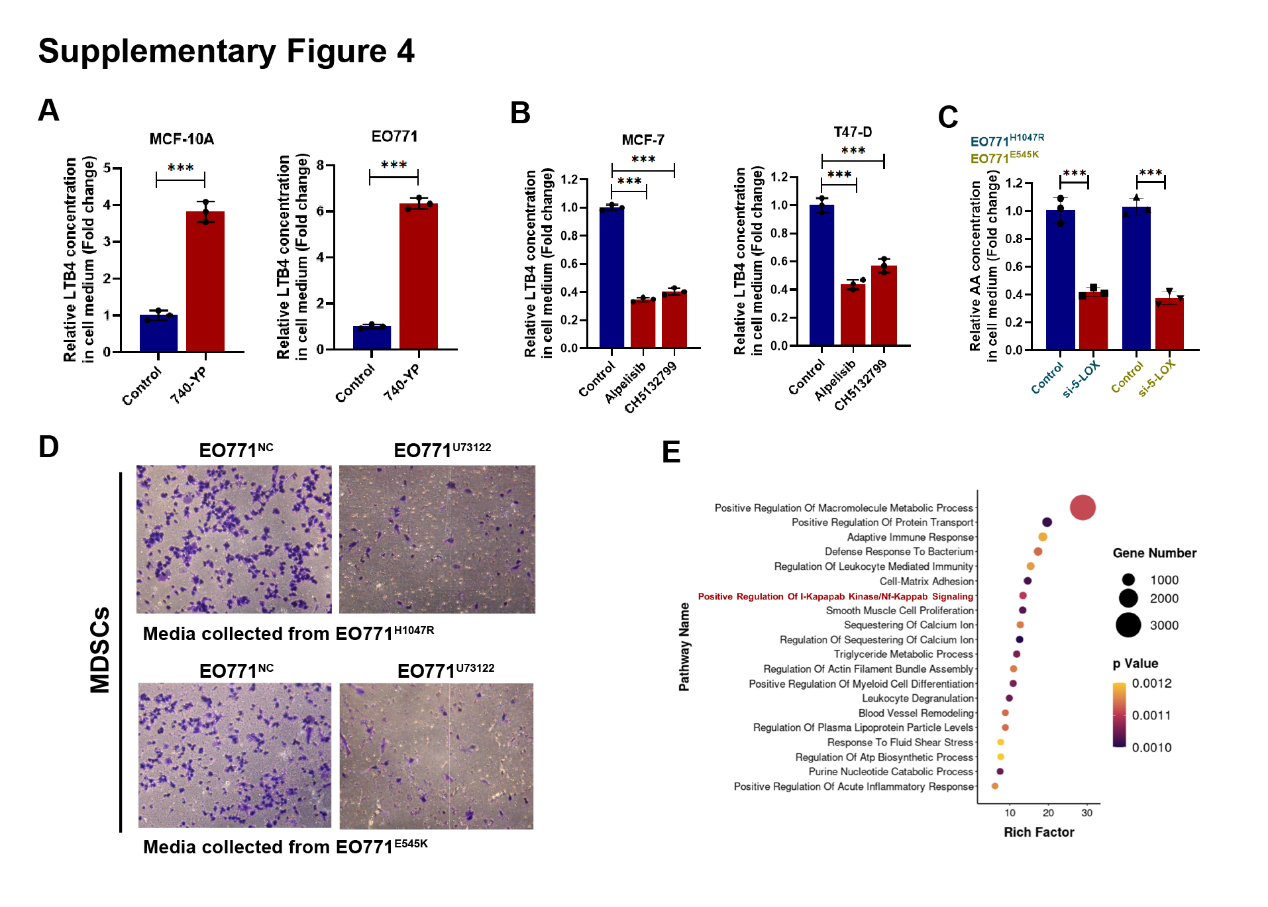


**Figure S4.** LTB4-dependent activation of NF-κB signaling pathway contributed to MDSCs recruitment in PIK3CAmut LBC. (A) The ELISA results of LTB4 from MCF-10A cell supernatant. (B) The ELISA results of LTB4 from MCF-7 and T-47D cell supernatants. (C) The ELISA results of AA. (D) The transwell chemotaxis assay *in vitro*. (E) KEGG shows the enrichment of NF-κB pathway in LTB4 treated MDSCs. Data represent mean ± SD, **P* < 0.05, ***P* < 0.01, ****P* < 0.001.


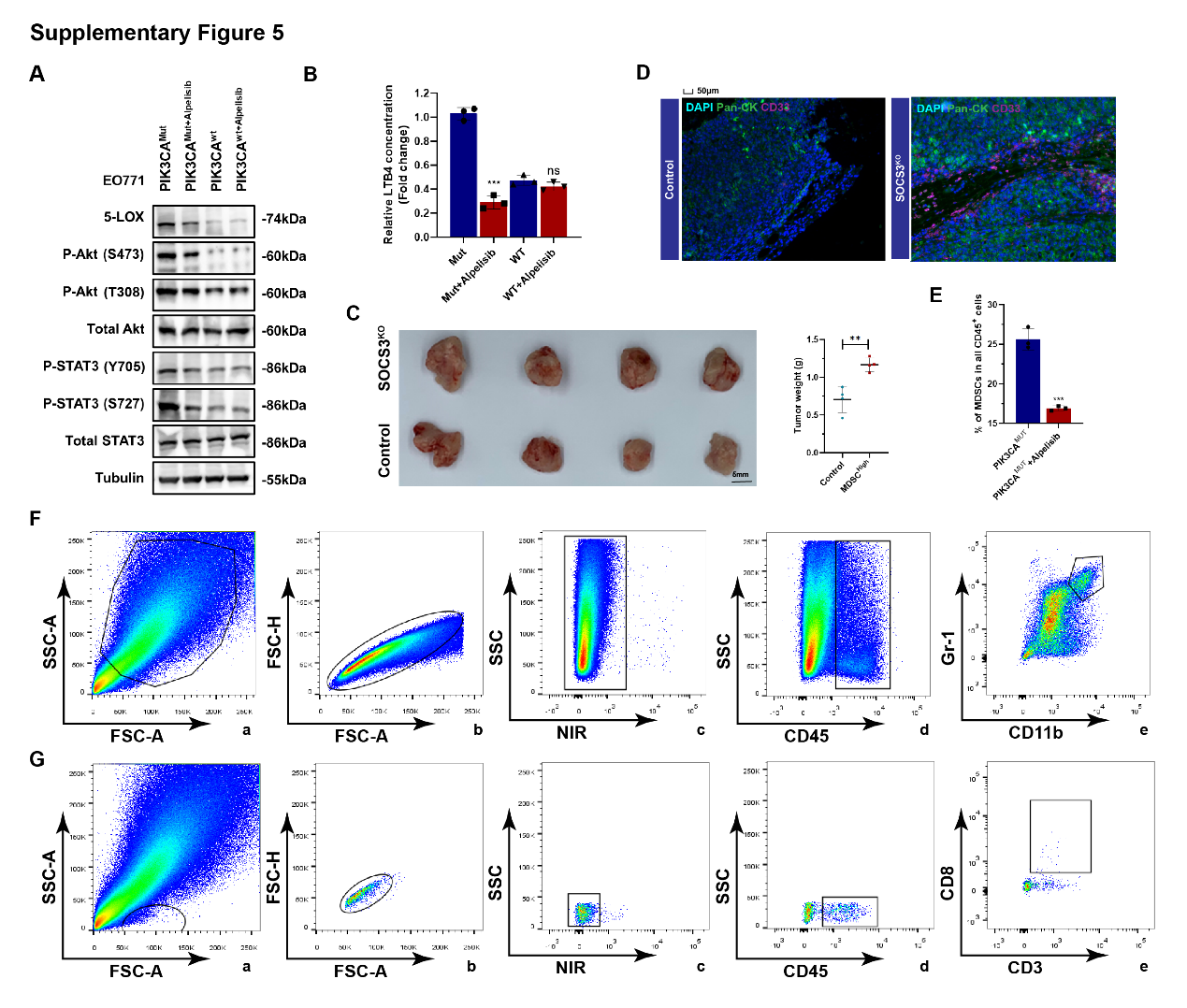


**Figure S5.** The suppressive TIME in PIK3CA^mut^ LBC xenografts was attenuated by PI3K inhibitor. (A) The protein levels of P-Akt, P-STAT3, and 5-LOX were measured by western blot. (B) The AA levels of the tumors measured by ELISA. (C) Tumor weight (g). (D) mIHC staining for MDSCs and tumor cells (E) Flow cytometry data showing the proportion of MDSCs. (F) The gating strategy for the flow cytometry analysis of MDSCs and the CD8^+^ T cells (G). Scale bar, 100 μm. Data represent mean ± SD, **P* < 0.05, ***P* < 0.01, ****P* < 0.001.

**
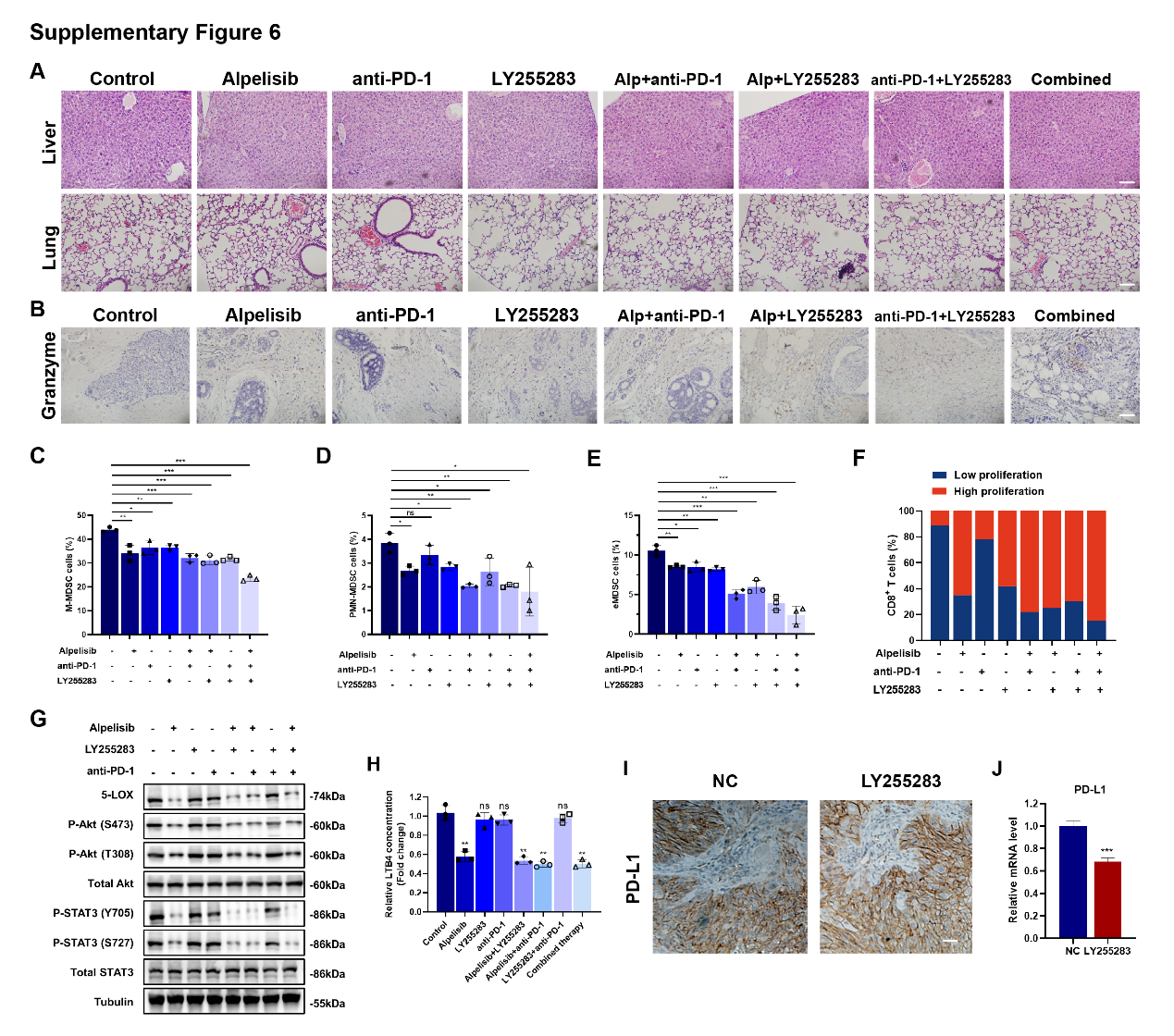
**

**Figure S6.** Targeted therapy against the PI3K/5-LOX/LTB4 axis combined with immunotherapy enhanced the antitumor efficacy *in vivo*. (A) Results of HE staining in lung tissues and liver tissues of mice. (B) IHC showed the expression of Granzyme. (C) The ratio of M-MDSCs, PMN-MDSCs (D) and eMDSCs (E) cells in the tumor tissues were measured by flow cytometry. (F) CFSE assay. (G) The protein levels of P-Akt, P-STAT3, and 5-LOX were measured by western blot. (H) The AA levels of the tumors measured by ELISA. (I) IHC showed the expression of PD-L1 in tumors. (J) mRNA level of PD-L1 in MDSCs. Data represent mean ± SD, **P* < 0.05, ***P* < 0.01, ****P* < 0.001.


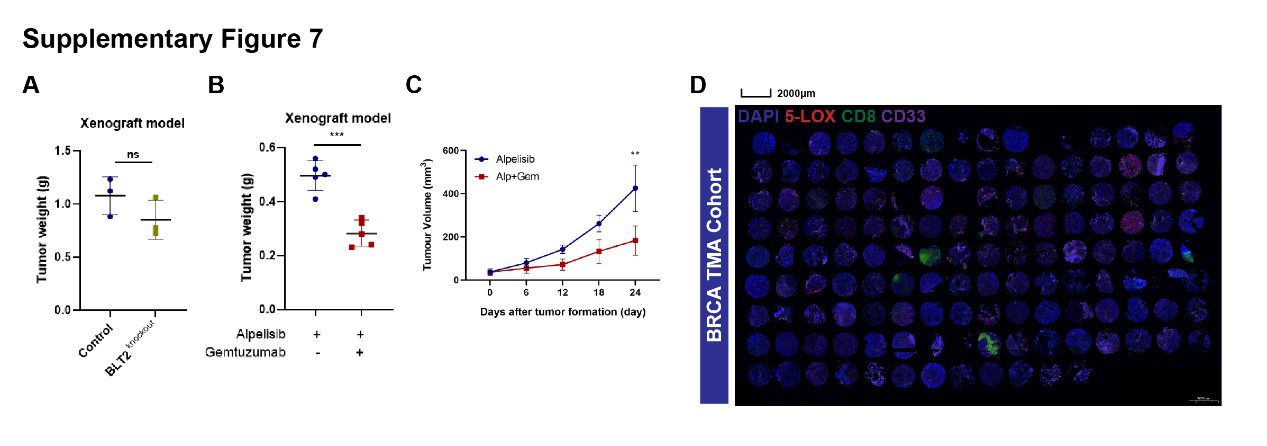


**Figure S7.** PIK3CA^mut^ induced AA metabolism pathway activation contribute to more effect on the MDSCs in the TIME. (A) Tumor weight (g). (B) Tumor weight (g) and tumor volume (C). (D) mIHC staining for 5-LOX, MDSCs, T cells and tumor cells. Data represent mean ± SD, **P* < 0.05, ***P* < 0.01, ****P* < 0.001.

**Supplementary Tables**

| **Table S1.** Clinical pathology information about TJMU patients. | |
| --- | --- |
| **Clinical parameters** | **N/total** |
| Total | 62 |
| Age |  |
| ≤50 years | 33 |
| >50 years | 29 |
| Tumor size |  |
| ≤2 cm | 17 |
| > 2 cm | 45 |
| Lymph nodes |  |
| Negative | 30 |
| Positive | 32 |
| Stage |  |
| I | 2 |
| II | 16 |
| III | 44 |
| Subtype |  |
| Luminal | 37 |
| Her2 positive | 17 |
| TNBC | 8 |
| TNBC: Triple-Negative Breast Cancer |  |

**Table S2.** Clinical pathology information about BRCA TMA.

| **Clinical parameters** | **N/total** |
| --- | --- |
| Total | 140 |
| Age |  |
| ≤50 years | 39 |
| >50 years | 101 |
| Tumor size |  |
| ≤2 cm | 33 |
| > 2 cm | 107 |
| Lymph nodes |  |
| Negative | 66 |
| Positive | 69 |
| Stage |  |
| I | 3 |
| II | 60 |
| III | 64 |
| Subtype |  |
| Luminal | 62 |
| Her2 positive | 43 |
| TNBC | 35 |
| TNBC: Triple-Negative Breast Cancer |  |

**Table S3.** Key resources table.

| **Reagent/Resource** | **Dilution** | **Applications** | **Source** | **Identifier** |
| --- | --- | --- | --- | --- |
| **Antibodies** | | | | |
| Total-AKT | 1:1000 | IB | Cell Signaling Technology | #4685 |
| Phospho-AKT (Ser473) | 1:1000 | IB | Cell Signaling Technology | #4060 |
| Phospho-AKT (Thr308) | 1:1000 | IB | Cell Signaling Technology | #13038 |
| Total-STAT3 | 1:1000 | IB | Cell Signaling Technology | #9139 |
| Phospho-Stat3 (Tyr705) | 1:1000; 1:100 | IB; ChIP | Cell Signaling Technology | #9145 |
| Phospho-Stat3 (Ser727) | 1:1000 | IB | Cell Signaling Technology | #49081 |
| 5-lipoxygenase (5-LO) | 1:1000; 1:200 | IB; mIHC | Cell Signaling Technology | #3289 |
| Phospho-IκB (Ser32) | 1:1000 | IB | Cell Signaling Technology | #2859 |
| CD33 | 1:500; 1:500 | IHC; mIHC | Abcam | #ab269456 |
| 5-lipoxygenase (5-LO) | 1:200 | IF | Abcam | #ab169755 |
| Pan-CK | 1:250 | mIHC | Abcam | #ab7753 |
| Tubulin | 1:5000 | IB | Abcam | #ab176560 |
| Histone H3 | 1:1000 | IB | Abcam | #ab1791 |
| CD11b | 1:200; 1:200 | IHC; mIHC | Abcam | #ab133357 |
| CD8 | 1:5000 | IHC; mIHC | Proteintech | #66868-1-Ig |
| NOX1 | 1:1000 | IB | Proteintech | #17772-1-AP |
| NOX4 | 1:1000 | IB | Proteintech | #14347-1-AP |
| NF-κB p65 | 1:1000 | IB | Proteintech | #10745-1-AP |
| IκB | 1:1000 | IB | Proteintech | #66418-1-Ig |
| BLT2 | 1:1000 | IB | Origene Technologies | TA351365S |
| Foxp3 | 1:200 | IHC | Bioss | #bs-10211R |
| CD163 | 1:200 | IHC | Bioss | #bs-2527R |
| **Chemicals** |  |  |  |  |
| Alpelisib |  |  | MedChemExpress | #HY-15744 |
| 740 Y-P |  |  | MedChemExpress | #HY-P0175 |
| LY255283 |  |  | MedChemExpress | #HY-15244 |
| LTB4 |  |  | Cayman Chemical | #20110 |
| U73122 |  |  | Selleck | #S8011 |
| Pembrolizumab |  |  | Selleck | #A2005 |
| CH5132799 |  |  | Selleck | #S2699 |
| **ELISA kits** |  |  |  |  |
| AA |  | ELISA | FineTest | EU2569 |
| 5-HETE |  | ELISA | FineTest | EU0185 |
| 12-HETE |  | ELISA | Detroit R&D | #12H1 |
| 15-HETE |  | ELISA | Detroit R&D | #15H1 |
| LTB4 |  | ELISA | Elabscience | E-EL-0061c |
| LTD4 |  | ELISA | Elabscience | E-EL-0113c |
| LTC4 |  | ELISA | MyBioSource | MBS2540381 |
| IFN-γ |  | ELISA | Bioss | Bsk12001 |
| IB: immunoblotting; IHC: immunohistochemistry; mIHC: multiplex immunohistochemistry; IF: immunofluorescence; ChIP: Chromatin immunoprecipitation; ELISA: Enzyme-linked immunosorbent assay | | | | |
|  | | | | |

| **Table S4.** Antibody list for CyTOF. | | | |
| --- | --- | --- | --- |
| **Antibodies** | **Tagged elements** | **Channel** | **Specificities** |
| CD45 | Pd | 102 | Mouse; Human |
| α-SMA | Pr | 141 | Mouse |
| CD23 | Ce | 142 | Mouse |
| CD11b | Nd | 143 | Mouse |
| PD-1 | Nd | 144 | Mouse; Human |
| CD69 | Nd | 145 | Mouse |
| CD8 | Nd | 146 | Mouse |
| NF-κB | Sm | 147 | Mouse |
| CD44 | Sm | 148 | Mouse |
| CD19 | Sm | 149 | Mouse |
| CD25 | Sm | 150 | Mouse |
| P-AKT | Eu | 151 | Mouse; Human |
| Ly-6G | Gd | 152 | Mouse |
| Foxp3 | Eu | 153 | Mouse |
| Vimentin | Gd | 154 | Mouse |
| CD83 | Gd | 155 | Mouse |
| P-mTOR | Gd | 156 | Mouse; Human |
| P-STAT3 | Gd | 158 | Mouse |
| F4/80 | Tb | 159 | Mouse |
| TNF-α | Dy | 160 | Mouse |
| Ki67 | Dy | 161 | Mouse |
| Ly-6C | Dy | 162 | Mouse |
| CD3 | Dy | 163 | Mouse; Human |
| CD14 | Dy | 164 | Mouse |
| IFN-γ | Ho | 165 | Mouse |
| IL-6 | Er | 166 | Mouse |
| CD163 | Er | 167 | Mouse |
| IL-10 | Tm | 169 | Mouse |
| CD86 | Yb | 170 | Mouse |
| CD138 | Yb | 171 | Mouse |
| CD4 | Yb | 172 | Mouse |
| CD117 | Yb | 173 | Mouse |
| ATG5 | Yb | 174 | Mouse |
| PD-L1 | Lu | 176 | Mouse |
| CD11c | Bi | 209 | Mouse |

**Table S5.** List of padlock sequence.

| **Name** | **Sequence (5’-3’)** |
| --- | --- |
| PIK3CA^E545K^ | AGCAGGAGAAAGATTTCTGCGAATAGCCATCCACTCCATTCTTCTGCGAATAGCCATCCACTCCATCTCTCTCTGAAATCACTA |
| PIK3CA^H1047R^ | ATGAAATACTCCAAAGCCTCCCTCGCATCAATACCGATCATCTCTTCCCCTCGCATCAATACCGATCATCGTGCATCATTCATTTGTTTC |

**Table S6.** List of qPCR primers sequence.

| **Primer name** | **Sequence (5’-3’)** |
| --- | --- |
| GAPDH | F: GGAGCGAGATCCCTCCAAAAT |
|  | R: GGCTGTTGTCATACTTCTCATGG |
| ALOX5 | F: ACAAGCCCTTCTACAACGACT |
|  | R: AGCTGGATCTCGCCCAGTT |

**Table S7.** List of ChIP-PCR primers sequence.

| **Primer name** | **Sequence (5’-3’)** |
| --- | --- |
| ALOX5-979/738 | F: GGTGAGAATGTAGGCAGCCG |
|  | R: CAAATGCCTGGAAGGGTGTG |
| ALOX5-584/390 | F: CAGGTCCAGCCAGTGAAGAA |
|  | R: CCTCTGCTCTCCCCAAGTTC |
| Negative Control | F: CATCTCAGCCCATTCCTGGC |
|  | R: GTCTCCTTGCTTTCCTACTGCT |

**Table S8.** Antibodies used in flow cytometry.

| **Antibodies** | **Company** | **Identifier** |
| --- | --- | --- |
| PerCP Rat Anti-Mouse CD45 | BD Biosciences | Cat#557235 |
| FITC Rat Anti-Mouse CD11b | BD Biosciences | Cat#557396 |
| PE Rat Anti-Mouse Gr-1 | BD Biosciences | Cat#553128 |
| PE Rat Anti-Mouse Ly6C | BD Biosciences | Cat#562728 |
| APC Rat Anti-Mouse Ly6G | BD Biosciences | Cat#565369 |
| PE-Cy5 Rat Anti-Mouse F4/80 | eBioscience | Cat#15-4801-82 |
| APC Mouse Anti-Mouse MHC II | BD Biosciences | Cat#562823 |
| FITC Rat Anti-Mouse CD3 | BD Biosciences | Cat#555274 |
| PE Rat Anti-Mouse CD8 | BD Biosciences | Cat#550798 |

**Table S9.** List of 33 enrichment pathways.

| **Pathway** |  |
| --- | --- |
| Focal Adhesion | |
| Gnrh Signaling Pathway | |
| Prion Diseases | |
| Natural Killer Cell Mediated Cytotoxicity | |
| Mapk Signaling Pathway | |
| Type I Diabetes Mellitus | |
| Melanogenesis | |
| Antigen Processing and Presentation | |
| Apoptosis | |
| Steroid Biosynthesis | |
| B Cell Receptor Signaling Pathway | |
| Leukocyte Transendothelial Migration | |
| Vascular Smooth Muscle Contraction | |
| Hematopoietic Cell Lineage | |
| Colorectal Cancer | |
| Wnt Signaling Pathway | |
| Prostate Cancer | |
| Chemokine Signaling Pathway | |
| Axon Guidance | |
| Regulation Of Actin Cytoskeleton | |
| Acute Myeloid Leukemia | |
| Arachidonic Acid Metabolism | |
| Oocyte Meiosis | |
| Calcium Signaling Pathway | |
| Viral Myocarditis | |
| Intestinal Immune Network for Iga Production | |
| Fc Epsilon Ri Signaling Pathway | |
| Primary Immunodeficiency | |
| T Cell Receptor Signaling Pathway | |
| Bladder Cancer | |
| Abc Transporters | |
| Complement And Coagulation Cascades | |
| Cell Cycle | |
